# Synthesis and fungicidal activity of novel 2,5-disubstituted-1,3,4- thiadiazole derivatives containing 5-phenyl-2-furan

**DOI:** 10.1038/srep20204

**Published:** 2016-01-29

**Authors:** Zi-Ning Cui, Ya-Sheng Li, De-Kun Hu, Hao Tian, Jia-Zhen Jiang, Yuan Wang, Xiao-Jing Yan

**Affiliations:** 1 Guangdong Province Key Laboratory of Microbial Signals and Disease Control, Department of Plant Pathology, South China Agricultural University, Guangzhou 510642, China; 2 Department of Applied Chemistry, College of Science, China Agricultural University, Beijing 100193, China; 3 Institute of Plant Protection, Chinese Academy of Agricultural Sciences, Beijing 100193, China

## Abstract

A series of 2,5-disubstituted-1,3,4-thiadiazoles were synthesized using Lawesson’s reagent by an efficient approach under microwave irradiation in good yields. Their structures were characterized by MS, IR, ^1^H NMR, ^13^C NMR, and elemental analysis. Their *in vitro* and *in vivo* fungicidal activities revealed that the title compounds exhibited considerable activity against five selected fungi, especially to *Phytophthora infestans*. In order to illustrate the mechanism of title compounds against *P. infestans*, scanning electron micrographs (SEM) and transmission electron micrographs (TEM) were applied. The morphological and ultrastructural studies demonstrated that compound **I18** led to swelling of hyphae, thickening and proliferating multilayer cell walls, excessive septation and accumulation of dense bodies. The bioassay results indicated compound **I18** might act on cell wall biosynthesis, and blocked the nutrition transportation and led to cells senescence and death. Meanwhile, compound **I18** had broad fungicidal activity against other twenty different kinds of fungi. These results suggested that title compounds were eligible to be development candidates and compound **I18** as a promising lead compound was worthy to be further discovery, especially against *P. infestans.*

1,3,4-Thiadiazoles are one of the prevalent and significant structural moieties^1^,^2^,^3^ in pharmaceuticals and agrochemicals having broad spectra of bioactivities, including anti-inflammatory^4^,^5^, antihypertensive^6^, antibacterial^7^, antituberculosis^8^, anticonvulsant^9^, antimicrobial^10^, antidepressants^11^, antileishmanial^12^, and anticancer^13^,^14^.

It has been reported that the compounds containing furan showed broad-spectrum bioactivities. A wide range of derivatives such as dibenzoylureas^15^, diacylhydrazines^16^,^17^,^18^,^19^,^20^,^21^, acylhydrazones^22^,^23^,^24^,^25^, semicarbazide^26^, pyrazole, 1,2,4-triazole^27^, 1,3,4-oxadiazole^28^, and carbamic acid esters^29^,^30^ containing 5-phenyl-2-furan moiety has been synthesized and studied in our group for the development of novel chemical entities as a lead molecule in drug and agrochemical discovery. All the compounds showed diverse and significant bioactivities such as fungicidal, insecticidal, and antitumor activities.

The standard method for the synthesis of 1,3,4-thiadiazoles involves cyclization of acylhydrazines (*N*,*N*-diacylhydrazines and monoacylhydrazines etc.) or thiohydrazines (thiosemicarbazides, bithioureas, and thiocarbazides etc.) employing usually POCl_3_^31^,^32^, PCl_5_^33^, FeCl_3_^34^ or H_2_SO_4_^35^ as dehydrating agents. The other important way is *via* the transformation of the oxygen atom in 1,3,4-oxadiazole, which acts as the bioisostere of 1,3,4-thiadiazole, to sulfur using P_2_S_5_^36^ and thiourea^37^. Thionation of acylhydrazines by the treatment of Lawesson’s reagent followed by cyclization and dehydrosulfurization was reported to produce 1,3,4-thiadiazoles in good yields^38^,^39^. Recently, there has been growing interests in the application of microwave irradiation in chemical reaction, the salient features being improved reaction rates and increased yields^28^,^40^,^41^.

In continuation of our research on the synthesis of biological heterocyclic compounds, a series of novel 1,3,4-thiadiazole derivatives containing 5-phenyl-2- furan moiety was synthesized by an efficient approach under microwave irradiation (Fig. 1). Their fungicidal activity was evaluated.

## Results and Discussion

### Synthesis

Synthesis of the title compounds 2,5-disubstituted-1,3,4-thiadiazoles (see supplementary information for ^1^H NMR and ^13^C NMR spectra) was achieved following a convenient procedure starting from commercially available 2-furoic acid and the substituted anilines as outlined in Fig. 2. 5-subsitituted phenyl-2-furoic acids **2** were prepared by the method of Meerwein arylation using copper (II)-catalyzed decomposition of diazonium salts. The diacylhydrazines **3** were obtained in moderate to good yields by the reaction of different 5-substituted phenyl-2-furoic chloride with differently substituted benzoylhydrazides in refluxing anhydrous dichloromethane.

Thionation of diacylhydrazines **3** with Lawesson’s reagent followed by oxidative cyclization in dry toluene led to the title compounds 1,3,4-thiadiazoles in good yields. Several selected one-pot microwave-assisted syntheses were carried out to establish the general validity check of the newly developed method. This method appeared to be economical and expeditious. The reaction proceeded well and smoothly under microwave irradiation within 15 min whereas 5–7 h was required under conventional reflux condition (Table 1). The yields were raised from 8.3% to 18.3% compared to the conventional method. This presented method, which was more facilitated and rapid than conventional method, indicated a good contribution to microwave-assisted synthetic methodologies.

The reaction of diacylhydrazines **3** with Lawesson’s reagent was also screened in different solvents including tetrahydrofuran, dioxane and xylene, but afforded less yields when compared to toluene.

### Fungicidal activity

*In vitro* fungicidal activities of title compounds against *P. infestans*, *V. mali*, *P. aspamgi*, *C. fulvum* Cke., *A. tenuis* Nees were listed in Table 2. The bioassay results showed that the title compounds had significant activities against the selected fungi. The comparison of the fungicidal activity of title compounds for five test fungi to those of positive control fungicides reached the following conclusions: (a) Compounds **I10**, **I18**, **I19**, **I25**, and **I31** exhibited excellent activity against *P. infestans*, and the EC_50_ values were 7.4, 5.7, 4.1, 8.4, and 18.1 μg/mL respectively, which were better than that of the commercial fungicides pyrimorph (EC_50_ = 25.2 μg mL^−1^) and hymexazol (EC_50_ = 29.1 μg mL^−1^). The preliminary structure-activity relationship showed that phenyl group without any substituent (R^1^ = H, such as compounds **I10**, **I18**, and **I25** or R^2^ = H, compound **I19**) was favored to the bioactivity. (b) For *V. mali*, compounds **I18** and **I21** showed considerable activity. Compound **I18** exhibited higher activity (EC_50_ = 9.7 μg mL^−1^) than that of control fungicides pyrimorph (EC_50_ = 32.5 μg mL^−1^) and hymexazol (EC_50_ = 10.9 μg mL^−1^). Compound **I21** had better activity (EC_50_ = 20.4 μg mL^−1^) than that of pyrimorph, but lower than that of hymexazol. (c) Compounds **I18** and **I20** had favorable fungicidal activity against *P. aspamgi* (EC_50_ = 21.7 and 23.1 μg mL^−1^) and *C. fulvum* (EC_50_ = 21.4 and 22.8 μg mL^−1^), which were better than that of pyrimorph, but lower than hymexazol. Besides that, compounds **I12** and **I29**, **I2** and **I5** showed significant activities against *P. aspamgi* and *C. fulvum* respectively. (d) All the compounds showed lower effect against *A. tenuis* except compound **I18**, which gave excellent activity and the EC_50_ value (5.8 μg mL^−1^) was better than that of pyrimorph (17.3 μg mL^−1^) and hymexazol (7.4 μg mL^−1^).

Due to the favorable *in vitro* bioactivity, *in vivo* activity against four fungi was also assessed and the results were presented in Table 3. Tendency of the results was in consistent with that of the *in vitro* bioactivity. Compounds **I10**, **I18**, **I19**, and **I25** exhibited a significant inhibition effect (exceeding 80% efficacy rate) against *P. infestans*, and the antifungal activities (control efficacy of 83.85 ± 1.85%, 84.21 ± 1.58, 87.15 ± 2.02 and 80.18 ± 2.01%) were better than that of both pyrimorph (77.15 ± 1.84%) and hymexazol (64.27 ± 1.72%) at 500 μg mL^−1^. For *C. fulvum*, it was worthy to note that compounds **I18** and **I20**, which efficacy rates were 77.14 ± 2.02% and 71.55 ± 1.20%, were found to be much more effective compared to the fungicide pyrimorph (46.21 ± 1.19%). Meanwhile, all the tested compounds were found safe for the plants.

**Figure 1:**
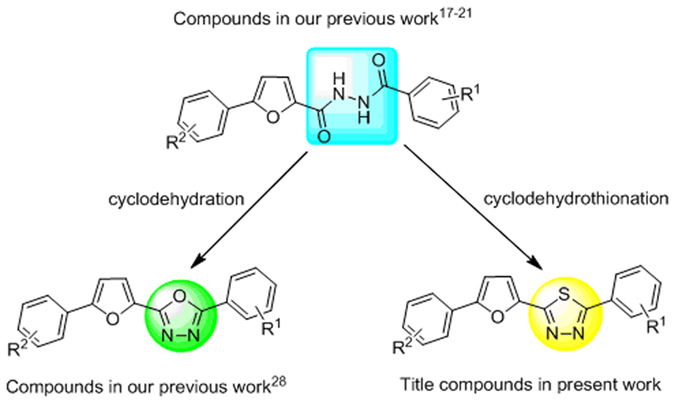
Design strategy for the title compounds.

**Figure 2:**
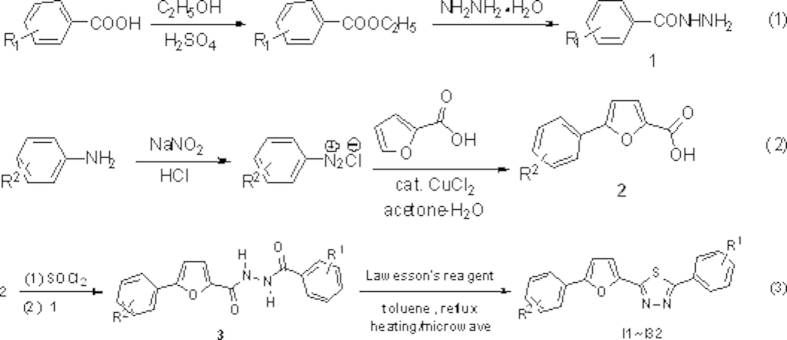
General synthetic procedure for title compounds.

Compared to the precursor diacylhydrazines **3** and cyclodehydrated compounds 1,3,4-oxadiazoles (Fig. 1), the title compounds exhibited improved and higher fungicidal activity to a certain extent. The fungicidal spectra were also broadened.

From the *in vitro* and *in vivo* bioassay results, it indicated that title compounds possessed significant fungicidal activities, especially against the *P. infestans*. Among them, compound **I18** gave great promise as a lead compound for further development. The fungicidal spectra against twenty fungi species of compound **I18** were evaluated and the results were listed in Table 4. The results revealed that compound **I18** had broad and excellent fungicidal activities, except against *T. roseum, B. cinerea*, and *P. melonis*.

### Effects of compound I18 on morphological and ultrastructural variation of *P. infestans*

Compound **I18** was effective in inhibiting mycelia radial growth on PDA medium (Table 2). Along the surface of culture media without compound **I18**, mycelium of *P. capsici* grew smoothly and uniformly. The whole colony appeared to be radiative from its centre and the rim of the colony was regular. However, in the media with compound **I18**, the growth of mycelium was seriously inhibited. The rim of the colony was changed to be irregular and concave-convex, and was not as smooth as that of blank control. Furthermore, high concentration of compound **I18** made this abnormal appearance much clear.

**Figure 3:**
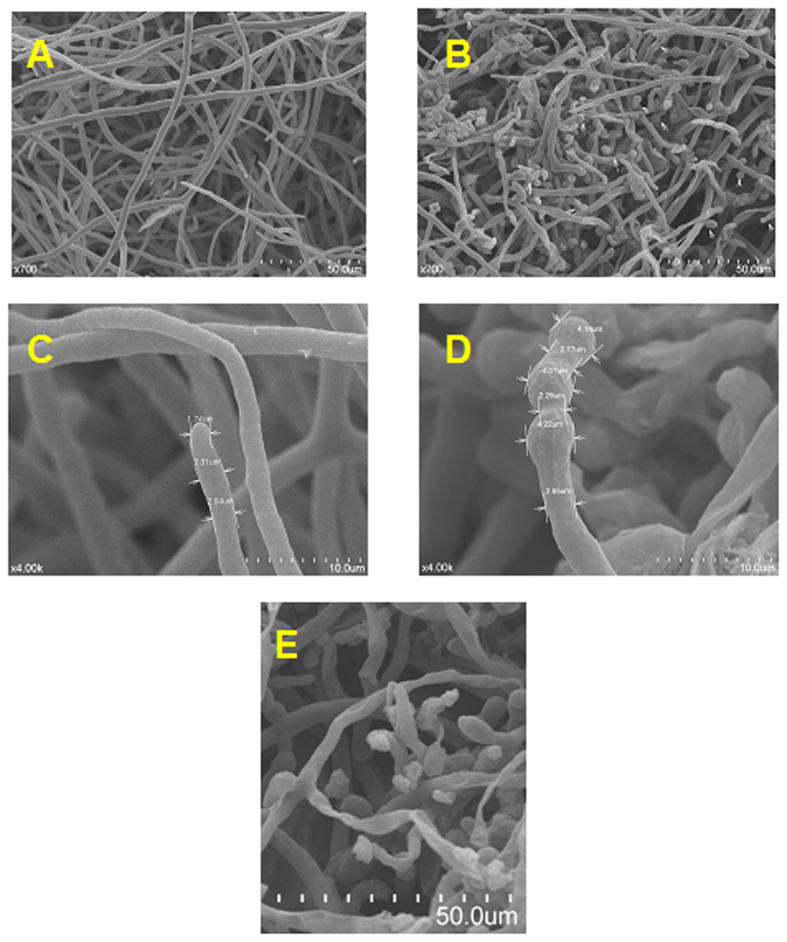
Scanning electron micrographs (SEM) of the hyphae from the colony of *P. infestans*: (**A,B,E**) 700×, bars, 50.0 μm; (**C,D**) 4000×, bars, 10.0 μm. (**A,C**) Sections of *P. infestans* hyphae were grown on PDA (blank control). (**B,D,E**) Sections of *P. infestans* hyphae were grown on PDA with 50 μg mL^−1^ compound **I18**.

Scanning electron micrographs (SEM) images of *P. infestans* treated with compound **I18** demonstrated the effects on the morphology of the hyphae (Fig. 3). SEM images revealed that the mycelium grew freshly and normally (the diameter was about 2.03 μm) in the culture media of blank control with low density and fine structure (Fig. 3A,C). However, in culture media with compound **I18** of 50 μg mL^−1^, mycelium grew abnormally with relatively high density of colony and some mycelia were entangled with each other. Some parts of the mycelium swelled (the diameter was about 4.14 μm) and distorted to form the “beaded” morphology on the tip, and others ruptured to produce shriveled and empty mycelia (Fig. 3B,E).

**Figure 4:**
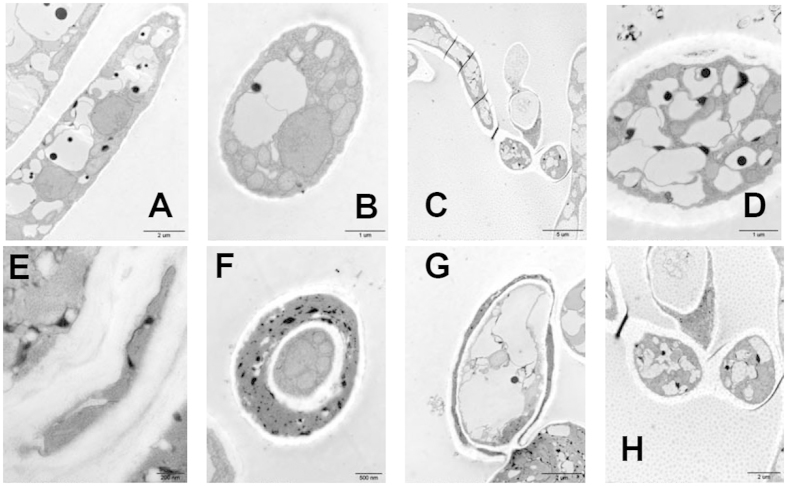
Transmission electron micrographs (TEM) of *P. infestans* hyphae in transversal (**A,C**) and longitudinal (**B,D–G**). (**A,B**) Sections of *P. infestans* hyphae were grown on PDA (blank control). (**C–G**) Sections of *P. infestans* hyphae were grown on PDA medium with 50 μg mL^−1^ compound **I18**.

*P. infestans* mycelial tip (5 mm) from the rim of an actively growing colony on PDA medium was investigated by TEM (Fig. 4). The blank control mycelia of *P. infestans* grown in the absence of compound **I18** demonstrated some cytological and ultrastructural features, which were typical vegetative hyphae of the genus^42^,^43^ (Fig. 4A,D). There were normal cell wall deposition and undulated plasmalemma, and cytoplasm containing vacuole and mitochondria were observed.

In the presence of compound **I18** at 50 μg mL^−1^, extensive cell wall thickening was the most conspicuous ultrastructural variation observed in hyphae (Fig. 4C,D). The number of vacuoles increased and vacuoles were distorted and disrupted under treatment with compound **I18** (3D). Vacuoles play an important role in mycelial growth, meanwhile with the function of maintaining fungal turgor pressure. The phenomena caused by compound **I18** were roughly the same as those caused by pyrimorph and dimethomorph, which had an effect on the biosynthesis of cell walls^43^,^44^,^45^. Hence, compound **I18** may retard fungal growth by acting on cell wall synthesis. The “beaded” hyphae were separated by the cell walls and false septa (Fig. 4C,H), which blocked the nutrition transportation and led to cells senescence and death. It revealed that the multilayer cell walls were formed and there was cytoplasmic substance in the interlayer of the proliferative cell walls (Fig. 4D–F). Gradually the cell walls exfoliated obviously and the cytoplasmic substance osmosis in the interlayer increased. Finally the cell wall ruptured and the cytoplasmic substance outflowed (Fig. 4F). That was the reason of producing shriveled and empty mycelia observed in SEM (Fig. 3B,E). Mitochondria and cell nuclear had the same appearance as blank control hyphae.

## Conclusions

In summary, we synthesized a series of 2-substituted phenyl-5-(5′-substituted phenyl-2′-furoyl)-1,3,4-thiadiazoles using Lawesson’s reagent by an efficient approach under microwave irradiation in good yields. The title compounds displayed significant fungicidal activity against various fungi, especially exhibited excellent fungicidal activity against *P. infestans*. Moreover, it was speculated that compound **I18** might act on the synthesis of cell walls from morphological and ultrastructural studies by SEM and TEM, which also revealed that compound **I18** could block the nutrition transportation and led to cells senescence and death. These results suggested that title compounds were eligible to be development candidates and compound **I18** as a promising lead compound was worthy to be further discovery, especially against *P. infestans.* However, further research should also be undergoing to confirm the specific mode of action of the title compounds.

## Materials and Methods

### Instruments

All the melting points were determined with a Cole-Parmer melting point apparatus (Cole-Parmer, Vernon Hills, Illinois, USA) while the thermometer was uncorrected. IR spectra were recorded on a Nicolet NEXUS-470 FTIR spectrometer (International Equipment Trading Ltd., Vernon Hills, Illinois, USA) with KBr pellets. ^1^H NMR and ^13^C NMR spectra were recorded with Bruker DPX400 (Bruker, Fallanden, Switzerland), while tetramethylsilane was used as an internal standard. Analytical thin-layer chromatography was carried out on silica gel 60 F254 plates, and spots were visualized with ultraviolet light. Mass spectra were measured on a Bruker APEX IV spectrometer (Bruker, Fallanden, Switzerland). Elemental analyses were performed on a Vario EL elemental analyzer. The microwave-assisted reaction was carried out with a CEM Microwave synthesizer (CEM Discover S-Class).

### Synthetic procedures

#### General synthetic procedure for the key intermediates

Intermediates 5-substituted phenyl-2-furoic acids **2** were synthesized from substituted aniline by Meerwein arylation reaction according to the reported literatures^25^,^26^,^27^,^28^,^29^. Intermediates diacylhydrazides **3** were synthesized using our previous procedure^17^,^28^.

#### General procedure for the synthesis of title compounds

A mixture of diacylhydrazines **3** (1 mmol) and Lawesson’s reagent (1.5 mmol) in toluene (10 mL) was refluxed for 5–7 h. After completion of the reaction as monitored by TLC, the reaction was quenched by addition of the saturated NaHCO_3_ solution. The organic layer was washed with brine, dried over MgSO_4_ and concentrated in vacuo. The residue was recrystalized from ethanol to afford pure products **I**.

#### General procedure for the synthesis of title compounds by microwave radiation

All reactions were carried out in a pressure tube, sealed with a Teflon septum. The mixture of the diacylhydraziness **3** (1 mmol) and Lawesson’s reagent (1.5 mmol) in toluene (20 mL) was taken in the pressure tube. The pressure tube was introduced to the centre of a CEM Discover microwave oven and irradiated for 15 min at 150 W (reaction temperature was set to 110 °C). After completion of the reaction, the reaction mixture was allowed to cool, and then, it was poured slowly with stirring into the saturated NaHCO_3_ solution. The organic layer was washed with brine, dried over MgSO_4_ and concentrated in vacuo. The residue was recrystalized from ethanol to afford pure products **I**.

### 2-(4-Methoxyphenyl)-5-(5-(4-nitrophenyl)furan-2-yl)-1,3,4-thiadiazole (I1)

Yellow solid, m.p. 238–239 °C; IR (KBr) *ν*_*max*_: 1603.52, 1513.85, 1434.78, 1411.64, 1335.46, 1257.36, 1172.51, 1106.94, 1030.77 cm^−1^. ^1^H NMR (400 MHz, CDCl_3_) 3.90 (s, 3H, OCH_3_), 7.02 (d, *J* = 8.8 Hz, 2H, PhH-Thia), 7.06 (d, *J* = 4.0 Hz, 1H, FuH), 7.33 (d, *J* = 3.6 Hz, 1H, FuH), 7.91 (d, *J* = 9.2 Hz, 2H, PhH-Fu), 7.97 (d, *J* = 8.4 Hz, 2H, PhH-Thia), 8.31 (d, *J* = 8.8 Hz, 2H, PhH-Fu). ^13^C NMR (100 MHz, CDCl_3_) *δ* 167.49 (C-2, Thia), 162.05 (C-4′, Ph-Thia), 156.59 (C-5, Thia), 153.44 (C-5, Fu), 146.99(C-4, Ph-Fu), 146.85 (C-2, Fu), 135.01 (C-1, Ph-Fu), 129.47 (2C, C-2′, C-6′, Ph-Thia), 124.45 (2C, C-2, C-6, Ph-Fu), 124.36 (2C, C-3, C-5, Ph-Fu), 122.27 (C-1′, Ph-Thia), 114.56 (2C, C-3′, C-5′, Ph-Thia), 113.52 (C-3, Fu), 111.19 (C-4, Fu), 55.42 (OCH_3_). ESIMS (*m*/*z*): 380.1 [M + H]^+^. Anal. Calcd. (%) for C_19_H_13_N_3_O_4_S: C, 60.15; H, 3.45; N, 11.08. Found: C, 59.98; H, 3.55; N, 11.17.

### 2-(4-Bromophenyl)-5-(5-(2-chlorophenyl)furan-2-yl)-1,3,4-thiadiazole (I2)

Yellow solid, m.p. 196–197 °C; IR (KBr) *ν*_*max*_: 3063.37, 1586.16, 1530.24, 1469.49, 1441.53, 1066.44, 1020.16 cm^−1^. ^1^H NMR (400 MHz, CDCl_3_) 7.26–7.32 (m, 2H, FuH + PhH-Fu), 7.35–7.38 (m, 2H, FuH + PhH-Fu), 7.49 (d, *J* = 8.0 Hz, 1H, PhH-Fu), 7.64 (d, *J* = 8.8 Hz, 2H, PhH-Thia), 7.88 (d, *J* = 8.4 Hz, 2H, PhH-Thia), 7.94 (dd, *J* = 1.4, 8.0 Hz, 1H, PhH-Fu). ^13^C NMR (100 MHz, CDCl_3_) *δ* 166.21 (C-2, Thia), 158.29 (C-5, Thia), 152.91 (C-5, Fu), 144.78 (C-2, Fu), 132.57 (2C, C-3′, C-5′, Ph-Thia), 131.09 (C-4, Ph-Fu), 130.89 (C-1, Ph-Fu), 129.37 (C-3, Ph-Fu), 129.31 (2C, C-2′, C-6′, Ph-Thia), 128.93 (C-1′, Ph-Thia), 128.37 (C-6, Ph-Fu), 128.07 (C-2, Ph-Fu), 127.19 (C-5, Ph-Fu), 125.73 (C-4′, Ph-Thia), 113.87 (C-3, Fu), 113.57 (C-4, Fu). ESIMS (*m/z*): 441.1 [M + Na]^+^. Anal. Calcd. (%) for C_18_H_10_BrClN_2_OS: C, 51.76; H, 2.41; N, 6.71. Found: C, 51.48; H, 2.63; N, 6.66.

### 2-(4-Methoxyphenyl)-5-(5-(4-chlorophenyl)furan-2-yl)-1,3,4-thiadiazole (I3)

Yellow solid, m.p. 169–170 °C; IR (KBr) *ν*_*max*_: 3092.30, 1606.41, 1518.67, 1480.10, 1264.11, 1171.54, 1091.51, 1019.19 cm^−1^. ^1^H NMR (400 MHz, CDCl_3_) 3.89 (s, 3H, OCH_3_), 6.82 (d, *J* = 3.6 Hz, 1H, FuH), 7.01 (d, *J* = 8.8 Hz, 2H, PhH-Thia), 7.27 (d, *J* = 3.6 Hz, 1H, FuH), 7.41 (d, *J* = 8.4 Hz, 2H, PhH-Fu), 7.69 (d, *J* = 8.8 Hz, 2H, PhH-Thia), 7.95 (d, *J* = 8.4 Hz, 2H, PhH-Fu). ^13^C NMR (100 MHz, CDCl_3_) *δ* 167.03 (C-2, Thia), 161.96 (C-4′, Ph-Thia), 157.18 (C-5, Thia), 155.11 (C-5, Fu), 145.32 (C-2, Fu), 134.33 (C-4, Ph-Fu), 129.51 (2C, C-2′, C-6′, Ph-Thia), 129.18 (2C, C-3, C-5, Ph-Fu), 128.11 (C-1, Ph-Fu), 125.54 (2C, C-2, C-6, Ph-Fu), 122.60 (C-1′, Ph-Thia), 114.64 (2C, C-3′, C-5′, Ph-Thia), 113.55 (C-3, Fu), 108.18 (C-4, Fu), 55.54 (OCH_3_). ESIMS (*m/z*): 369.1 [M + H]^+^. Anal. Calcd. (%) for C_19_H_13_ClN_2_O_2_S: C, 61.87; H, 3.55; N, 7.60. Found: C, 62.09; H, 3.23; N, 7.89.

### 2-(5-(4-Chlorophenyl)furan-2-yl)-5-(2-methoxyphenyl)-1,3,4-thiadiazole (I4)

Yellow solid, m.p. 160–161 °C; IR (KBr) *ν*_*max*_: 3411.46, 1599.66, 1536.02, 1478.17, 1432.85, 1298.82, 1249.65, 1164.79, 1093.44, 1050.05, 1020.16 cm^−1^. ^1^H NMR (400 MHz, CDCl_3_) 4.07 (s, 3H, OCH_3_), 6.82 (d, *J* = 3.6 Hz, 1H, FuH), 7.07 (dd, *J* = 3.2, 8.4 Hz, 1H, PhH-Thia), 7.14 (t, *J* = 7.6 Hz, 1H, PhH-Thia), 7.28 (d, *J* = 3.6 Hz, 1H, FuH), 7.40–7.42 (m, 2H, PhH-Fu), 7.47–7.51 (m, 1H, PhH-Thia), 7.70–7.73 (m, 2H, PhH-Fu), 8.55 (dd, *J* = 1.6, 8.0 Hz, 1H, PhH-Thia). ^13^C NMR (100 MHz, CDCl_3_) *δ* 160.88 (C-2, Thia), 158.98 (C-5, Thia), 155.79 (C-2′, Ph-Thia), 154.85 (C-5, Fu), 145.90 (C-2, Fu), 134.10 (C-4, Ph-Fu), 132.11 (C-4′, Ph-Thia), 129.08 (2C, C-3, C-5, Ph-Fu), 128.74 (C-6′, Ph-Thia), 128.25 (C-1, Ph-Fu), 125.50 (2C, C-2, C-6, Ph-Fu), 121.35 (C-5′, Ph-Thia), 119.02 (C-1′, Ph-Thia), 113.12 (C-3, Fu), 111.30 (C-3′, Ph-Thia), 108.08 (C-4, Fu), 55.80 (OCH_3_). ESIMS (*m/z*): 369.2 [M + H]^+^. Anal. Calcd. (%) for C_19_H_13_ClN_2_O_2_S: C, 61.87; H, 3.55; N, 7.60. Found: C, 61.62; H, 3.74; N, 7.43.

### 2-(3-Chlorophenyl)-5-(5-(4-chlorophenyl)furan-2-yl)-1,3,4-thiadiazole (I5)

Yellow solid, m.p. 178–179 °C; IR (KBr) *ν*_*max*_: 3048.91, 1684.52, 1573.63, 1536.99, 1479.13, 1432.85, 1093.44 cm^−1^. ^1^H NMR (400 MHz, CDCl_3_) 6.84 (d, *J* = 3.6 Hz, 1H, FuH), 7.33 (d, *J* = 3.6 Hz, 1H, FuH), 7.41–7.50 (m, 4H, 2PhH-Thia + 2PhH-Fu), 7.70 (d, *J* = 8.0 Hz, 2H, PhH-Fu), 7.89 (d, *J* = 7.2 Hz, 1H, PhH-Thia), 8.03 (s, 1H, PhH-Thia). ^13^C NMR (100 MHz, CDCl_3_) *δ* 165.59 (C-2, Thia), 158.36 (C-5, Thia), 155.55 (C-5, Fu), 144.98 (C-2, Fu), 135.29 (C-1′, Ph-Thia), 134.54 (C-4, Ph-Fu), 131.52 (C-3′, Ph-Thia), 131.10 (C-5′, Ph-Thia), 130.50 (C-6′, Ph-Thia), 129.20 (2C, C-3, C-5, Ph-Fu), 127.93 (C-1, Ph-Fu), 127.76 (C-4′, Ph-Thia), 125.99 (C-2′, Ph-Thia), 125.59 (2C, C-2, C-6, Ph-Fu), 114.16 (C-3, Fu), 108.24 (C-4, Fu). ESIMS (*m/z*): 395.1 [M + Na]^+^. Anal. Calcd. (%) for C_18_H_10_Cl_2_N_2_OS: C, 57.92; H, 2.70; N, 7.51. Found: C, 58.14; H, 2.81; N, 7.29.

### 2-(5-(4-Bromophenyl)furan-2-yl)-5-(4-methoxyphenyl)-1,3,4-thiadiazole (I6)

Yellow solid, m.p. 201–202 °C; IR (KBr) *ν*_*max*_: 3068.41, 1621.32, 1543.52, 1497.42, 1454.21, 1272.43, 1175.25, 1102.24, 1053.21 cm^−1^. ^1^H NMR (400 MHz, CDCl_3_) 3.89 (s, 3H, OCH_3_), 6.83 (d, *J* = 3.6 Hz, 1H, FuH), 7.01 (d, *J* = 8.8 Hz, 2H, PhH-Thia), 7.26 (d, *J* = 3.6 Hz, 1H, FuH), 7.56 (d, *J* = 8.8 Hz, 2H, PhH-Fu), 7.63 (d, *J* = 8.8 Hz, 2H, PhH-Fu), 7.95 (d, *J* = 9.2 Hz, 2H, PhH-Thia). ^13^C NMR (100 MHz, CDCl_3_) *δ* 166.98 (C-2, Thia), 161.95 (C-4′, Ph-Thia), 157.16 (C-5, Thia), 155.06 (C-5, Fu), 145.28 (C-2, Fu), 132.03 (2C, C-3, C-5, Ph-Fu), 129.44 (2C, C-2′, C-6′, Ph-Thia), 128.44 (C-1, Ph-Fu), 125.69 (2C, C-2, C-6, Ph-Fu), 122.51 (C-1′, Ph-Thia), 122.44 (C-4, Ph-Fu), 114.56 (2C, C-3′, C-5′, Ph-Thia), 113.49 (C-3, Fu), 108.20 (C-4, Fu), 55.46 (OCH_3_). ESIMS (*m/z*): 437.1[M + Na]^+^. Anal. Calcd. (%) for C_19_H_13_BrN_2_O_2_S: C, 55.22; H, 3.17; N, 6.78. Found: C, 55.01; H, 3.42; N, 6.59.

### 2-(4-Chlorophenyl)-5-(5-(2-chlorophenyl)furan-2-yl)-1,3,4-thiadiazole (I7)

Light yellow solid, m.p. 195–196 °C; IR (KBr) *ν*_*max*_: 3031.71, 1693.12, 1565.92, 1547.87, 1481.21, 1475.85, 1089.49, 1021.67 cm^−1^. ^1^H NMR (400 MHz, CDCl_3_) 7.27–7.31 (m, 1H, PhH-Fu), 7.32 (d, *J* = 3.6 Hz, 1H, FuH), 7.36 (d, *J* = 3.6 Hz, 1H, FuH), 7.37–7.41 (m, 1H, PhH-Fu), 7.47–7.50 (m, 3H, 2PhH-Thia + PhH-Fu), 7.94–7.98 (m, 3H, 2PhH-Thia + PhH-Fu). ^13^C NMR (100 MHz, CDCl_3_) δ 166.03 (C-2, Thia), 158.18 (C-5, Thia), 152.79 (C-5, Fu), 144.68 (C-2, Fu), 137.28 (C-4′, Ph-Thia), 130.98 (C-4, Ph-Fu), 130.79 (C-1, Ph-Fu), 129.51 (2C, C-3′, C-5′, Ph-Thia), 129.26 (C-3, Ph-Fu), 129.04 (2C, C-2′, C-6′, Ph-Thia), 128.39 (C-1′, Ph-Thia), 128.27 (C-6, Ph-Fu), 127.97 (C-2, Ph-Fu), 127.08 (C-5, Ph-Fu), 113.74 (C-3, Fu), 113.46 (C-4, Fu). ESIMS (*m/z*): 373.1 [M + H]^+^. Anal. Calcd. (%) for C_18_H_10_Cl_2_N_2_OS: C, 57.92; H, 2.70; N, 7.51. Found: C, 58.25; H, 2.49; N, 7.76.

### 2-(2-Chlorophenyl)-5-(5-(2-chlorophenyl)furan-2-yl)-1,3,4-thiadiazole (I8)

Light yellow solid, m.p. 145–146 °C; IR (KBr) *ν*_*max*_: 3079.52, 1651.43, 1574.96, 1514.64, 1492.31, 1445.76, 1046.49, 1015.34 cm^−1^. ^1^H NMR (400 MHz, CDCl_3_) 7.27–7.31 (m, 1H, PhH-Fu), 7.33–7.48 (m, 6H, 2FuH + 2PhH-Thia + 2PhH-Fu), 7.55–7.57 (m, 1H, PhH-Fu), 7.98–8.00 (m, 1H, PhH-Thia), 8.40–8.43 (m, 1H, PhH-Thia). ^13^C NMR (100 MHz, CDCl_3_) *δ* 162.53 (C-2, Thia), 159.54 (C-5, Thia), 152.74 (C-5, Fu), 144.76 (C-2, Fu), 132.47 (C-1′, Ph-Thia), 131.65 (C-4′, Ph-Thia), 131.17 (C-3′, Ph-Thia), 130.94 (C-4, Ph-Fu), 130.74 (C-1, Ph-Fu), 130.61 (C-6′, Ph-Thia), 129.19 (C-3, Ph-Fu), 128.78 (C-2′, Ph-Thia), 128.33 (C-6, Ph-Fu), 128.02 (C-2, Ph-Fu), 127.43 (C-5′, Ph-Thia), 127.10 (C-5, Ph-Fu), 113.75 (C-3, Fu), 113.47 (C-4, Fu). ESIMS (*m/z*): 373.2 [M + H]^+^. Anal. Calcd. (%) for C_18_H_10_Cl_2_N_2_OS: C, 57.92; H, 2.70; N, 7.51. Found: C, 57.76; H, 2.81; N, 7.83.

### 2-(5-(2-Chlorophenyl)furan-2-yl)-5-(4-ethoxyphenyl)-1,3,4-thiadiazole (I9)

Yellow solid, m.p. 163–164 °C; IR (KBr) *ν*_*max*_: 3103.43, 1646.32, 1575.36, 1492.60, 1278.82, 1229.43, 1182.47, 1071.43, 1021.34 cm^−1^. ^1^H NMR (400 MHz, CDCl_3_) 1.45 (t, *J* = 6.8 Hz, 3H, C-CH_3_), 4.11 (q, *J* = 6.8 Hz, 2H, O-CH_2_-C), 6.98 (d, *J* = 8.0 Hz, 2H, PhH-Thia), 7.25–7.31 (m, 3H, 2FuH + PhH-Fu), 7.36–7.39 (m, 1H, PhH-Fu), 7.47 (d, *J* = 8.4 Hz, 1H, PhH-Fu), 7.92–7.96 (m, 3H, 2PhH-Thia + PhH-Fu). ^13^C NMR (100 MHz, CDCl_3_) *δ* 167.20 (C-2, Thia), 161.39 (C-4′, Ph-Thia), 157.17 (C-5, Thia), 152.36 (C-5, Fu), 144.97 (C-2, Fu), 130.93 (C-4, Ph-Fu), 130.66 (C-1, Ph-Fu), 129.47 (2C, C-2′, C-6′, Ph-Thia), 129.08 (C-3, Ph-Fu), 128.22 (C-6, Ph-Fu), 128.06 (C-2, Ph-Fu), 127.06 (C-5, Ph-Fu), 122.35 (C-1′, Ph-Thia), 115.03 (2C, C-3′, C-5′, Ph-Thia), 113.39 (C-3, Fu), 113.18 (C-4, Fu), 63.75 (OCH_2_), 14.71 (CH_3_). ESIMS (*m/z*): 383.1 [M + H]^+^. Anal. Calcd. (%) for C_20_H_15_ClN_2_O_2_S: C, 62.74; H, 3.95; N, 7.32. Found: C, 62.99; H, 4.09; N, 7.61.

### 2-(5-(2-Chlorophenyl)furan-2-yl)-5-phenyl-1,3,4-thiadiazole (I10)

Light yellow solid, m.p. 139–140 °C; IR (KBr) *ν*_*max*_: 3093.82, 1646.32, 1607.62, 1575.36, 1492.60, 1427.32, 1264.92, 1229.43, 1182.47, 1071.43, 1017.51 cm^−1^. ^1^H NMR (400 MHz, CDCl_3_) 7.28–7.30 (m, 1H, PhH-Fu), 7.31 (d, *J* = 3.6 Hz, 1H, FuH), 7.34 (d, *J* = 3.6 Hz, 1H, FuH), 7.36–7.40 (m, 1H, PhH-Fu), 7.47–7.51 (m, 4H, 2PhH-Thia + 2PhH-Fu), 7.95 (dd, *J* = 1.2, 8.0 Hz, 1H, PhH-Thia), 8.00–8.03 (m, 2H, PhH-Thia). ^13^C NMR (100 MHz, CDCl_3_) *δ* 167.31 (C-2, Thia), 157.99 (C-5, Thia), 152.62 (C-5, Fu), 144.88 (C-2, Fu), 131.18 (C-1′, Ph-Thia), 130.96 (C-4, Ph-Fu), 130.75 (C-1, Ph-Fu), 129.92 (C-4′, Ph-Thia), 129.22 (2C, C-2′, C-6′, Ph-Thia), 129.18 (C-3, Ph-Fu), 128.28 (C-6, Ph-Fu), 128.05 (C-2, Ph-Fu), 127.93 (2C, C-3′, C-5′, Ph-Thia), 127.08 (C-5, Ph-Fu), 113.50 (C-3, Fu), 113.43 (C-4, Fu). ESIMS (*m/z*): 339.1 [M + H]^+^. Anal. Calcd. (%) for C_18_H_11_ClN_2_OS: C, 63.81; H, 3.27; N, 8.27. Found: C, 63.65; H, 3.56; N, 8.02.

### 2-(4-Chlorophenyl)-5-(5-(2,4-difluorophenyl)furan-2-yl)-1,3,4-thiadiazole (I11)

Yellow solid, m.p. 192–193 °C; IR (KBr) *ν*_*max*_: 1679.24, 1609.67, 1584.53, 1499.87, 1453.83, 1434.42, 1421.73, 1225.62, 1158.15, 1095.83, 1016.74 cm^−1^. ^1^H NMR (400 MHz, CDCl_3_) 6.92–7.03 (m, 3H, FuH + 2PhH-Fu), 7.33 (d, *J* = 3.6 Hz, 1H, FuH), 7.48 (d, *J* = 8.4 Hz, 2H, PhH-Thia), 7.88–7.96 (m, 3H, 2PhH-Thia + PhH-Fu). ^13^C NMR (100 MHz, CDCl_3_) *δ* 165.99 (C-2, Thia), 162.62 (dd, ^1^*J*_C-F_ = 247.2 Hz, ^3^*J*_C-F_ = 8.7 Hz, C-4, Ph-Fu), 159.23 (dd, ^1^*J*_C-F_ = 253.1 Hz, ^3^*J*_C-F_ = 11.4 Hz, C-2, Ph-Fu), 158.05 (C-5, Thia), 149.97 (C-5, Fu), 144.51 (C-2, Fu), 137.33 (C-4′, Ph-Thia), 129.53 (2C, C-3′, C-5′, Ph-Thia), 129.04 (2C, C-2′, C-6′, Ph-Thia), 128.33 (C-1′, Ph-Thia), 127.38 (dd, ^3^*J*_C-F_ = 9.6 Hz, ^3^*J*_C-F_ = 4.4 Hz, C-6, Ph-Fu), 114.54 (dd, ^2^*J*_C-F_ = 12.0 Hz, ^4^*J*_C-F_ = 3.9 Hz, C-1, Ph-Fu), 114.26 (C-3, Fu), 112.04 (d, ^4^*J*_C-F_ = 3.6 Hz, C-4, Fu), 112.01 (dd, ^2^*J*_C-F_ = 21.4 Hz, ^4^*J*_C-F_ = 3.6 Hz, C-5, Ph-Fu), 104.77 (dd, ^2^*J*_C-F_ = 25.4 Hz, ^2^*J*_C-F_ = 25.4 Hz, C-3, Ph-Fu). ESIMS (*m/z*): 397.1 [M + Na]^+^. Anal. Calcd. (%) for C_18_H_9_ClF_2_N_2_OS: C, 57.68; H, 2.42; N, 7.47. Found: C, 57.33; H, 2.70; N, 7.19.

### 2-(2-Chlorophenyl)-5-(5-(2,4-difluorophenyl)furan-2-yl)-1,3,4-thiadiazole (I12)

Light yellow solid, m.p. 124–125 °C; IR (KBr) *ν*_*max*_: 1668.74, 1606.46, 1534.46, 1434.42, 1403.62, 1401.41, 1231.69, 1072.63, 1089.49 cm^−1^. ^1^H NMR (400 MHz, CDCl_3_) 6.92–7.04 (m, 3H, FuH + 2PhH-Fu), 7.37 (d, *J* = 3.6 Hz, 1H, FuH), 7.44–7.47 (m, 2H, PhH-Thia), 7.55–7.58 (m, 1H, PhH-Thia), 7.93–7.99 (m, 1H, PhH-Fu), 8.41–8.43 (m, 1H, PhH-Thia). ^13^C NMR (100 MHz, CDCl_3_) *δ* 162.55 (dd, ^1^*J*_C-F_ = 250.8 Hz, ^3^*J*_C-F_ = 12.4 Hz, C-4, Ph-Fu), 162.42 (C-2, Thia), 159.37 (C-5, Thia), 159.19 (dd, ^1^*J*_C-F_ = 252.2 Hz, ^3^*J*_C-F_ = 10.6 Hz, C-2, Ph-Fu), 149.87 (C-5, Fu), 144.66 (C-2, Fu), 132.44 (C-1′, Ph-Thia), 131.65 (C-4′, Ph-Thia), 131.14 (C-3′, Ph-Thia), 130.61 (C-6′, Ph-Thia), 128.76 (C-2′, Ph-Thia), 127.44 (dd, ^3^*J*_C-F_ = 9.5 Hz, ^3^*J*_C-F_ = 4.3 Hz, C-6, Ph-Fu), 127.43 (C-5′, Ph-Thia), 114.58 (dd, ^2^*J*_C-F_ = 12.0 Hz, ^4^*J*_C-F_ = 3.9 Hz, C-1, Ph-Fu), 114.13 (C-3, Fu), 112.05 (d, ^4^*J*_C-F_ = 3.6 Hz, C-4, Fu), 112.02 (dd, ^2^*J*_C-F_ = 21.4 Hz, ^4^*J*_C-F_ = 3.6 Hz, C-5, Ph-Fu), 104.71 (dd, ^2^*J*_C-F_ = 25.4 Hz, ^2^*J*_C-F_ = 25.4 Hz, C-3, Ph-Fu). ESIMS (*m/z*): 375.1 [M + H]^+^. Anal. Calcd. (%) for C_18_H_9_ClF_2_N_2_OS: C, 57.68; H, 2.42; N, 7.47. Found: C, 57.96; H, 2.67; N, 7.21.

### 2-(5-(2,4-Difluorophenyl)furan-2-yl)-5-(*p*-tolyl)-1,3,4-thiadiazole (I13)

Yellow solid, m.p. 172–173 °C; IR (KBr) *ν*_*max*_: 3121.15, 2912.65, 1630.87, 1555.83, 1478.71, 1457.92, 1415.57, 1279.53, 1118.69, 1080.87, 1037.93, 1010.14 cm^−1^. ^1^H NMR (400 MHz, CDCl_3_) 2.43 (s, 3H, CH_3_), 6.91–7.03 (m, 3H, FuH + 2PhH-Fu), 7.30–7.32 (m, 3H, FuH + 2PhH-Thia), 7.89–7.95 (m, 3H, 2PhH-Thia + PhH-Fu). ^13^C NMR (100 MHz, CDCl_3_) *δ* 167.41 (C-2, Thia), 162.58 (dd, ^1^*J*_C-F_ = 250.6 Hz, ^3^*J*_C-F_ = 12.2 Hz, C-4, Ph-Fu), 159.24 (dd, ^1^*J*_C-F_ = 255.7 Hz, ^3^*J*_C-F_ = 14.3 Hz, C-2, Ph-Fu), 157.48 (C-5, Thia), 149.63 (C-5, Fu), 144.79 (C-2, Fu), 141.74 (C-4′′, Ph-Thia), 129.90 (2C, C-3′, C-5′, Ph-Thia), 127.83 (2C, C-2′, C-6′, Ph-Thia), 127.38 (dd, ^3^*J*_C-F_ = 9.5 Hz, ^3^*J*_C-F_ = 4.3 Hz, C-6, Ph-Fu), 127.13 (C-1′, Ph-Thia), 114.64 (dd, ^2^*J*_C-F_ = 12.0 Hz, ^4^*J*_C-F_ = 3.9 Hz, C-1, Ph-Fu), 113.81 (C-3, Fu), 112.02 (d, ^4^*J*_C-F_ = 3.6 Hz, C-4, Fu), 112.00 (dd, ^2^*J*_C-F_ = 21.3 Hz, ^4^*J*_C-F_ = 3.6 Hz, C-5, Ph-Fu), 104.71 (dd, ^2^*J*_C-F_ = 25.4 Hz, ^2^*J*_C-F_ = 25.4 Hz, C-3, Ph-Fu), 21.53 (CH_3_). ESIMS (*m/z*): 355.2 [M + H]^+^. Anal. Calcd. (%) for C_19_H_12_F_2_N_2_OS: C, 64.40; H, 3.41; N, 7.91. Found: C, 64.14; H, 3.36; N, 7.79.

### 2-(5-(2,4-Difluorophenyl)furan-2-yl)-5-(4-methoxyphenyl)-1,3,4-thiadiazole (I14)

Yellow solid, m.p. 200–201 °C; IR (KBr) *ν*_*max*_: 1668.17, 1578.45, 1498.31, 1457.92, 1272.43, 1186.32, 1162.21, 1072.27 cm^−1^. ^1^H NMR (400 MHz, CDCl_3_) 3.89 (s, 3H, OCH_3_), 6.91–6.97 (m, 2H, FuH + PhH-Fu), 6.98–7.02 (m, 3H, 2PhH-Thia + PhH-Fu), 7.29 (d, *J* = 3.6 Hz, 1H, FuH), 7.89–7.96 (m, 3H, 2PhH-Thia + PhH-Fu). ^13^C NMR (100 MHz, CDCl_3_) *δ* 167.05 (C-2, Thia), 162.55 (dd, ^1^*J*_C-F_ = 250.4 Hz, ^3^*J*_C-F_ = 12.2 Hz, C-4, Ph-Fu), 161.98 (C-4′, Ph-Thia), 159.21 (dd, ^1^*J*_C-F_ = 254.1 Hz, ^3^*J*_C-F_ = 12.5 Hz, C-2, Ph-Fu), 157.07 (C-5, Thia), 149.55 (C-5, Fu), 144.83 (C-2, Fu), 129.46 (2C, C-2′, C-6′, Ph-Thia), 127.35 (dd, ^3^*J*_C-F_ = 9.5 Hz, ^3^*J*_C-F_ = 4.3 Hz, C-6, Ph-Fu), 122.53 (C-1′, Ph-Thia), 114.67 (dd, ^2^*J*_C-F_ = 12.0 Hz, ^4^*J*_C-F_ = 3.9 Hz, C-1, Ph-Fu), 114.58 (2C, C-3′, C-5′, Ph-Thia), 113.63 (C-3, Fu), 112.03 (d, ^4^*J*_C-F_ = 3.6 Hz, C-4, Fu), 112.01 (dd, ^2^*J*_C-F_ = 21.4 Hz, ^4^*J*_C-F_ = 3.6 Hz, C-5, Ph-Fu), 104.69 (dd, ^2^*J*_C-F_ = 25.4 Hz, ^2^*J*_C-F_ = 25.4 Hz, C-3, Ph-Fu), 55.47 (OCH_3_). ESIMS (*m/z*): 371.3 [M + H]^+^. Anal. Calcd. (%) for C_19_H_12_F_2_N_2_O_2_S: C, 61.61; H, 3.27; N, 7.56. Found: C, 61.47; H, 3.54; N, 7.41.

### 2-(5-(4-Fluorophenyl)furan-2-yl)-5-(*m*-tolyl)-1,3,4-thiadiazole (I15)

Yellow solid, m.p. 150–151 °C; IR (KBr) *ν*_*max*_: 1674.65, 1599.38, 1485.52, 1438.45, 1402.97, 1282.53, 1176.32, 1084.21, 1026.43, 1008.25 cm^−1^. ^1^H NMR (400 MHz, CDCl_3_) 2.44 (s, 3H, CH_3_), 6.76 (d, *J* = 3.6 Hz, 1H, FuH), 7.13 (t, *J* = 8.6 Hz, 2H, PhH-Fu), 7.28–7.32 (m, 2H, FuH + PhH-Thia), 7.38 (t, *J* = 7.4 Hz, 1H, PhH-Thia), 7.72–7.84 (m, 4H, 2PhH-Thia + 2PhH-Fu). ^13^C NMR (100 MHz, CDCl_3_) *δ* 167.23 (C-2, Thia), 162.77 (d, ^1^*J*_C-F_ = 247.6 Hz, C-4, Ph-Fu), 157.86 (C-5, Thia), 155.41 (C-5, Fu), 144.89 (C-2, Fu), 139.04 (C-3′, Ph-Thia), 131.92 (C-2′, Ph-Thia), 129.72 (C-1′, Ph-Thia), 129.06 (C-5′, Ph-Thia), 128.33 (C-4′, Ph-Thia), 126.12 (2C, d, ^3^*J*_C-F_ = 8.2 Hz, C-2, C-6, Ph-Fu), 125.87 (d, ^4^*J*_C-F_ = 3.3 Hz, C-1, Ph-Fu), 125.07 (C-6′, Ph-Thia), 115.97 (2C, d, ^2^*J*_C-F_ = 21.3 Hz, C-3, C-5, Ph-Fu), 113.73 (C-3, Fu), 107.41 (C-4, Fu), 21.31 (CH_3_). ESIMS (*m/z*): 337.1 [M + H]^+^. Anal. Calcd. (%) for C_19_H_13_FN_2_OS: C, 67.84; H, 3.90; N, 8.33. Found: C, 68.09; H, 4.17; N, 8.14.

### 2-(2-Chlorophenyl)-5-(5-(3-fluorophenyl)furan-2-yl)-1,3,4-thiadiazole (I16)

Light yellow solid, m.p. 154–155 °C; IR (KBr) *ν*_*max*_: 1689.53, 1617.42, 1592.42, 1503.87, 1489.23, 1464.22, 1421.73, 1227.52, 1155.15, 1085.83, 1019.74 cm^−1^. ^1^H NMR (400 MHz, CDCl_3_) 6.88 (d, *J* = 3.6 Hz, 1H, FuH), 7.05 (td, *J* = 2.0, 8.0 Hz, 1H, PhH-Fu), 7.35 (d, *J* = 3.2 Hz, 1H, FuH), 7.39–7.50 (m, 4H, 2PhH-Thia + 2PhH-Fu), 7.55–7.58 (m, 2H, PhH-Thia + PhH-Fu), 8.40–8.43 (m, 1H, PhH-Thia). ^13^C NMR (100 MHz, CDCl_3_) *δ* 163.15 (d, ^1^*J*_C-F_ = 244.6 Hz, C-3, Ph-Fu), 162.51(C-2, Thia), 159.49 (C-5, Thia), 155.21 (d, ^4^*J*_C-F_ = 3.1 Hz, C-5, Fu), 145.30 (C-2, Fu), 132.50 (C-1′, Ph-Thia), 131.68 (C-4′, Ph-Thia), 131.50 (d, ^3^*J*_C-F_ = 8.5 Hz, C-1, Ph-Fu), 131.16 (C-3′, Ph-Thia), 130.64 (C-6′, Ph-Thia), 130.59 (d, ^3^*J*_C-F_ = 9.8 Hz, C-5, Ph-Fu), 128.77 (C-2′, Ph-Thia), 127.44 (C-5′, Ph-Thia), 120.06 (d, ^4^*J*_C-F_ = 2.9 Hz, C-6, Ph-Fu), 115.46 (d, ^2^*J*_C-F_ = 21.3 Hz, C-2, Ph-Fu), 113.89 (C-3, Fu), 111.26 (d, ^2^*J*_C-F_ = 23.6 Hz, C-4, Ph-Fu), 108.74 (C-4, Fu). ESIMS (*m/z*): 357.1 [M + H]^+^. Anal. Calcd. (%) for C_18_H_10_ClFN_2_OS: C, 60.59; H, 2.82; N, 7.85. Found: C, 60.34; H, 3.01; N, 7.64.

### 2-(5-(4-Fluorophenyl)furan-2-yl)-5-(*p*-tolyl)-1,3,4-thiadiazole (I17)

Yellow solid, m.p. 190–191 °C; IR (KBr) *ν*_*max*_: 1666.35, 1599.26, 1527.48, 1495.29, 1452.27, 1426.73, 1281.37, 1182.92, 1094.11, 1016.43 cm^−1^. ^1^H NMR (400 MHz, CDCl_3_) 2.43 (s, 3H, CH_3_), 6.77 (d, *J* = 3.6 Hz, 1H, FuH), 7.14 (t, *J* = 8.6 Hz, 2H, PhH-Fu), 7.28 (d, *J* = 3.6 Hz, 1H, FuH), 7.30 (d, *J* = 8.0 Hz, 2H, PhH-Thia), 7.73–7.77 (m, 2H, PhH-Fu), 7.90 (d, *J* = 8.0 Hz, 2H, PhH-Thia). ^13^C NMR (100 MHz, CDCl_3_) *δ* 167.25 (C-2, Thia), 162.83 (d, ^1^*J*_C-F_ = 247.7 Hz, C-4, Ph-Fu), 157.71 (C-5, Thia), 155.46 (C-5, Fu), 145.02 (C-2, Fu), 141.69 (C-4′, Ph-Thia), 129.92 (2C, C-3′, C-5′, Ph-Thia), 127.83 (2C, C-2′, C-6′, Ph-Thia), 127.20 (C-1′, Ph-Thia), 126.23 (2C, d, ^3^*J*_C-F_ = 8.2 Hz, C-2, C-6, Ph-Fu), 125.97 (d, ^4^*J*_C-F_ = 3.3 Hz, C-1, Ph-Fu), 116.04 (2C, d, ^2^*J*_C-F_ = 21.9 Hz, C-3, C-5, Ph-Fu), 113.70 (C-3, Fu), 107.45 (C-4, Fu), 21.54 (CH_3_). ESIMS (*m/z*): 337.2 [M + H]^+^. Anal. Calcd. (%) for C_19_H_13_FN_2_OS: C, 67.84; H, 3.90; N, 8.33. Found: C, 67.59; H, 3.67; N, 8.62.

### 2-(5-(4-Fluorophenyl)furan-2-yl)-5-phenyl-1,3,4-thiadiazole (I18)

Yellow solid, m.p. 178–179 °C; IR (KBr) *ν*_*max*_: 1683.25, 1616.32, 1583.46, 1497.63, 1454.12, 1437.65, 1284.92, 1239.63, 1192.42, 1081.23, 1014.21 cm^−1^. ^1^H NMR (400 MHz, CDCl_3_) 6.77 (d, *J* = 3.6 Hz, 1H, FuH), 7.14 (t, *J* = 8.6 Hz, 2H, PhH-Fu), 7.30 (d, *J* = 4.0 Hz, 1H, FuH), 7.50–7.52 (m, 3H, PhH-Thia), 7.73–7.77 (m, 2H, PhH-Fu), 8.00–8.03 (m, 2H, PhH-Thia). ^13^C NMR (100 MHz, CDCl_3_) *δ* 167.08 (C-2, Thia), 162.85 (d, ^1^*J*_C-F_ = 247.7 Hz, C-4, Ph-Fu), 158.06 (C-5, Thia), 155.57 (C-5, Fu), 144.95 (C-2, Fu), 131.15 (C-1′, Ph-Thia), 129.93 (C-4′, Ph-Thia), 129.23 (2C, C-2′, C-6′, Ph-Thia), 127.91 (2C, C-3′, C-5′, Ph-Thia), 126.25 (2C, d, ^3^*J*_C-F_ = 8.2 Hz, C-2, C-6, Ph-Fu), 125.94 (d, ^4^*J*_C-F_ = 3.4 Hz, C-1, Ph-Fu), 116.05 (2C, d, ^2^*J*_C-F_ = 22.0 Hz, C-3, C-5, Ph-Fu), 113.84 (C-3, Fu), 107.46 (C-4, Fu). ESIMS (*m/z*): 323.1 [M + H]^+^. Anal. Calcd. (%) for C_18_H_11_FN_2_OS: C, 67.07; H, 3.44; N, 8.69. Found: C, 67.28; H, 3.21; N, 8.86.

### 2-(4-Chlorophenyl)-5-(5-phenylfuran-2-yl)-1,3,4-thiadiazole (I19)

Yellow solid, m.p. 185–186 °C; IR (KBr) *ν*_*max*_: 1612.52, 1573.42, 1447.33, 1434.32, 1417.25, 1244.22, 1219.33, 1061.43, 1021.52 cm^−1^. ^1^H NMR (400 MHz, CDCl_3_) 6.84 (d, *J* = 3.6 Hz, 1H, FuH), 7.32 (d, *J* = 3.6 Hz, 1H, FuH), 7.33–7.37 (m, 1H, PhH-Fu), 7.42–7.48 (m, 4H, 2PhH-Thia + 2PhH-Fu), 7.76–7.78 (m, 2H, PhH-Fu), 7.94 (d, *J* = 8.4 Hz, 2H, PhH-Thia). ^13^C NMR (100 MHz, CDCl_3_) *δ* 165.71 (C-2, Thia), 158.33 (C-5, Thia), 156.58 (C-5, Fu), 144.74 (C-2, Fu), 137.15 (C-4′, Ph-Thia), 129.47 (2C, C-3′, C-5′, Ph-Thia), 129.44 (C-1, Ph-Fu), 128.98 (2C, C-2′, C-6′, Ph-Thia), 128.90 (2C, C-3, C-5, Ph-Fu), 128.68 (C-4, Ph-Fu), 128.45 (C-1′, Ph-Thia), 124.33 (2C, C-2, C-6, Ph-Fu), 113.96 (C-3, Fu), 107.81 (C-4, Fu). ESIMS (*m/z*): 339.1 [M + H]^+^. Anal. Calcd. (%) for C_18_H_11_ClN_2_OS: C, 63.81; H, 3.27; N, 8.27. Found: C, 64.12; H, 3.49; N, 7.98.

### 2-(2-Chlorophenyl)-5-(5-phenylfuran-2-yl)-1,3,4-thiadiazole (I20)

Yellow solid, m.p. 133–134 °C; IR (KBr) *ν*_*max*_: 1612.52, 1573.42, 1532.38, 1439.83, 1424.12, 1410.16, 1224.22, 1209.43, 1031.23, 1012.57 cm^−1^. ^1^H NMR (400 MHz, CDCl_3_) 6.86 (d, *J* = 3.2 Hz, 1H, FuH), 7.34–7.38 (m, 2H, FuH + PhH-Fu), 7.44–7.47 (m, 4H, 2PhH-Thia + 2PhH-Fu), 7.54–7.58 (m, 1H, PhH-Thia), 7.79–7.81 (m, 2H, PhH-Fu), 8.40–8.42 (m, 1H, PhH-Thia). ^13^C NMR (100 MHz, CDCl_3_) *δ* 162.24 (C-2, Thia), 159.72 (C-5, Thia), 156.58 (C-5, Fu), 144.85 (C-2, Fu), 132.44 (C-1′, Ph-Thia), 131.56 (C-4′, Ph-Thia), 131.13 (C-3′, Ph-Thia), 130.60 (C-6′, Ph-Thia), 129.50 (C-1, Ph-Fu), 128.88 (2C, C-3, C-5, Ph-Fu), 128.85 (C-2′, Ph-Thia), 128.62 (C-4, Ph-Fu), 127.40 (C-5′, Ph-Thia), 124.37 (2C, C-2, C-6, Ph-Fu), 113.94 (C-3, Fu), 107.77 (C-4, Fu). ESIMS (*m/z*): 339.2 [M + H]^+^. Anal. Calcd. (%) for C_18_H_11_ClN_2_OS: C, 63.81; H, 3.27; N, 8.27. Found: C, 63.64; H, 3.51; N, 8.36.

### 2-(5-(4-Methoxyphenyl)furan-2-yl)-5-(*m*-tolyl)-1,3,4-thiadiazole (I21)

Yellow solid, m.p. 123–124 °C; IR (KBr) *ν*_*max*_: 1602.63, 1556.22, 1507.38, 1468.37, 1422.45, 1297.62, 1219.65, 1154.79, 1073.64, 1011.16 cm^−1^. ^1^H NMR (400 MHz, CDCl_3_) 2.45 (s, 3H, CH_3_), 3.86 (s, 3H, OCH_3_), 6.70 (d, *J* = 3.6 Hz, 1H, FuH), 6.96 (d, *J* = 8.8 Hz, 2H, PhH-Fu), 7.27–7.32 (m, 2H, FuH + PhH-Thia), 7.36–7.40 (m, 1H, PhH-Thia), 7.70 (d, *J* = 8.8 Hz, 2H, PhH-Fu), 7.78 (d, *J* = 7.6 Hz, 1H, PhH-Thia), 7.84 (s, 1H, PhH-Thia). ^13^C NMR (100 MHz, CDCl_3_) *δ* 166.96 (C-2, Thia), 159.97 (C-5, Thia), 158.18 (C-4, Ph-Fu), 156.58 (C-5, Fu), 144.30 (C-2, Fu), 139.04 (C-3′, Ph-Thia), 131.83 (C-2′, Ph-Thia), 129.88 (C-1′, Ph-Thia), 129.06 (C-5′, Ph-Thia), 128.36 (C-4′, Ph-Thia), 125.88 (C-6′, Ph-Thia), 125.09 (2C, C-2, C-6, Ph-Fu), 122.52 (C-1, Ph-Fu), 114.32 (2C, C-3, C-5, Ph-Fu), 113.87 (C-3, Fu), 106.24 (C-4, Fu), 55.37 (OCH_3_), 21.35 (CH_3_). ESIMS (*m/z*): 371.2[M + Na]^+^. Anal. Calcd. (%) for C_20_H_16_N_2_O_2_S: C, 68.94; H, 4.63; N, 8.04. Found: C, 69.18; H, 4.47; N, 8.33.

### 2-(4-Chlorophenyl)-5-(5-(*p*-tolyl)furan-2-yl)-1,3,4-thiadiazole (I22)

Brown solid, m.p. 211–212 °C; IR (KBr) *ν*_*max*_: 1639.26, 1576.02, 1498.87, 1462.85, 1425.37, 1288.42, 1250.65, 1134.79, 1040.05, 1013.13 cm^−1^. ^1^H NMR (400 MHz, CDCl_3_) 6.78 (d, *J* = 3.2 Hz, 1H, FuH), 7.25 (d, *J* = 8.4 Hz, 2H, PhH-Fu), 7.31 (d, *J* = 3.2 Hz, 1H, FuH), 7.47 (d, *J* = 8.8 Hz, 2H, PhH-Thia), 7.66 (d, *J* = 8.0 Hz, 2H, PhH-Fu), 7.95 (d, *J* = 8.8 Hz, 2H, PhH-Thia). ^13^C NMR (100 MHz, CDCl_3_) *δ* 165.57 (C-2, Thia), 158.44 (C-5, Thia), 156.91 (C-5, Fu), 144.36 (C-2, Fu), 138.82 (C-4, Ph-Fu), 137.11 (C-4′, Ph-Thia), 129.59 (2C, C-3, C-5, Ph-Fu), 129.46 (2C, C-3′, C-5′, Ph-Thia), 128.98 (2C, C-2′, C-6′, Ph-Thia), 128.49 (C-1′, Ph-Thia), 126.77 (C-1, Ph-Fu), 124.32 (2C, C-2, C-6, Ph-Fu), 114.05 (C-3, Fu), 107.15 (C-4, Fu), 21.41 (CH_3_). ESIMS (*m/z*): 353.1 [M + H]^+^. Anal. Calcd. (%) for C_19_H_13_ClN_2_OS: C, 64.68; H, 3.71; N, 7.94. Found: C, 64.49; H, 3.92; N, 7.76.

### 2-(5-(2-Fluorophenyl)furan-2-yl)-5-(4-methoxyphenyl)-1,3,4-thiadiazole (I23)

Yellow solid, m.p. 171–172 °C; IR (KBr) *ν*_*max*_: 1719.16, 1673.19, 1566.92, 1503.58, 1468.47, 1418.69, 1273.12, 1240.45, 1124.69, 1020.06, 1011.43 cm^−1^. ^1^H NMR (400 MHz, CDCl_3_) 7.00–7.03 (m, 3H, FuH + 2PhH-Thia), 7.14–7.19 (m, 1H, PhH-Fu), 7.24–7.28 (m, 1H, PhH-Fu), 7.29–7.35 (m, 2H, FuH + PhH-Fu), 7.92–7.97 (m, 3H, 2PhH-Thia + PhH-Fu). ^13^C NMR (100 MHz, CDCl_3_) *δ* 167.06 (C-2, Thia), 161.97 (C-4′, Ph-Thia), 159.02 (d, ^1^*J*_C-F_ = 250.4 Hz, C-2, Ph-Fu), 157.27 (C-5, Thia), 150.37 (d, ^4^*J*_C-F_ = 3.1 Hz, C-5, Fu), 144.84 (C-2, Fu), 129.57 (d, ^3^*J*_C-F_ = 8.5 Hz, C-4, Ph-Fu), 129.48 (2C, C-2′, C-6′, Ph-Thia), 126.32 (d, ^4^*J*_C-F_ = 2.6 Hz, C-4, Fu), 124.51 (d, ^4^*J*_C-F_ = 3.5 Hz, C-5, Ph-Fu), 122.58 (C-1′, Ph-Thia), 117.98 (d, ^2^*J*_C-F_ = 11.8 Hz, C-1, Ph-Fu), 116.14 (d, ^2^*J*_C-F_ = 21.3 Hz, C-3, Ph-Fu), 114.59 (2C, C-3′, C-5′, Ph-Thia), 113.65 (C-3, Fu), 112.65 (d, ^3^*J*_C-F_ = 12.2 Hz, C-6, Ph-Fu), 55.49 (OCH_3_). ESIMS (*m/z*): 375.2 [M + Na]^+^. Anal. Calcd. (%) for C_19_H_13_FN_2_O_2_S: C, 64.76; H, 3.72; N, 7.95. Found: C, 64.76; H, 3.72; N, 7.95.

### 2-(5-(2-Fluorophenyl)furan-2-yl)-5-(*m*-tolyl)-1,3,4-thiadiazole (I24)

Yellow solid, m.p. 130–131 °C; IR (KBr) *ν*_*max*_: 1699.27, 1664.38, 1576.32, 1498.87, 1472.15, 1421.97, 1283.72, 1254.35, 1184.29, 1109.27, 1025.16 cm^−1^. ^1^H NMR (400 MHz, CDCl_3_) 7.03 (t, *J* = 3.6 Hz, 1H, FuH), 7.14–7.19 (m, 1H, PhH-Fu), 7.23–7.32 (m, 4H, FuH + PhH-Thia + 2PhH-Fu), 7.36–7.40 (m, 1H, PhH-Thia), 7.79 (d, *J* = 7.6 Hz, 1H, PhH-Thia), 7.85 (s, 1H, PhH-Thia), 7.92–7.96 (m, 1H, PhH-Fu). ^13^C NMR (100 MHz, CDCl_3_) *δ* 167.43 (C-2, Thia), 159.01 (d, ^1^*J*_C-F_ = 250.5 Hz, C-2, Ph-Fu), 157.85 (C-5, Thia), 150.49 (d, ^4^*J*_C-F_ = 3.1 Hz, C-5, Fu), 144.76 (C-2, Fu), 139.07 (C-3′, Ph-Thia), 131.96 (C-2′, Ph-Thia), 129.75 (C-1′, Ph-Thia), 129.61 (d, ^3^*J*_C-F_ = 8.4 Hz, C-4, Ph-Fu), 129.08 (C-5′, Ph-Thia), 128.39 (C-4′, Ph-Thia), 126.30 (d, ^4^*J*_C-F_ = 2.6 Hz, C-4, Fu), 125.13 (C-6′, Ph-Thia), 124.50 (d, ^4^*J*_C-F_ = 3.5 Hz, C-5, Ph-Fu), 117.90 (d, ^2^*J*_C-F_ = 11.8 Hz, C-1, Ph-Fu), 116.18 (d, ^2^*J*_C-F_ = 21.2 Hz, C-3, Ph-Fu), 113.84 (C-3, Fu), 112.65 (d, ^3^*J*_C-F_ = 12.2 Hz, C-6, Ph-Fu), 21.33 (CH_3_). ESIMS (*m/z*): 337.2 [M + H]^+^. Anal. Calcd. (%) for C_19_H_13_FN_2_OS: C, 67.84; H, 3.90; N, 8.33. Found: C, 67.59; H, 4.13; N, 8.17.

### 2-(5-(2-Fluorophenyl)furan-2-yl)-5-phenyl-1,3,4-thiadiazole (I25)

Yellow solid, m.p. 112–113 °C; IR (KBr) *ν*_*max*_: 1689.46, 1598.27, 1556.02, 1489.37, 1452.21, 1415.74, 1271.51, 1261.75, 1124.81, 1070.45, 1012.83 cm^−1^. ^1^H NMR (400 MHz, CDCl_3_) 7.03 (t, *J* = 3.6 Hz, 1H, FuH), 7.15–7.19 (m, 1H, PhH-Fu), 7.24–7.28 (m, 1H, PhH-Fu), 7.30–7.34 (m, 1H, PhH-Fu), 7.35 (d, *J* = 3.6 Hz, 1H, FuH), 7.49–7.52 (m, 3H, PhH-Thia), 7.95 (td, *J* = 1.6, 7.6 Hz, 1H, PhH-Fu), 8.01–8.03 (m, 2H, PhH-Thia). ^13^C NMR (100 MHz, CDCl_3_) *δ* 167.22 (C-2, Thia), 159.05 (d, ^1^*J*_C-F_ = 250.5 Hz, C-2, Ph-Fu), 158.00 (C-5, Thia), 150.58 (d, ^4^*J*_C-F_ = 3.1 Hz, C-5, Fu), 144.76 (C-2, Fu), 131.16 (C-1′, Ph-Thia), 129.94 (C-4′, Ph-Thia), 129.66 (d, ^3^*J*_C-F_ = 8.4 Hz, C-4, Ph-Fu), 129.23 (2C, C-2′, C-6′, Ph-Thia), 127.93 (2C, C-3′, C-5′, Ph-Thia), 126.34 (d, ^4^*J*_C-F_ = 2.6 Hz, C-4, Fu), 124.52 (d, ^4^*J*_C-F_ = 3.5 Hz, C-5, Ph-Fu), 117.94 (d, ^2^*J*_C-F_ = 11.7 Hz, C-1, Ph-Fu), 116.17 (d, ^2^*J*_C-F_ = 21.2 Hz, C-3, Ph-Fu), 113.95 (C-3, Fu), 112.69 (d, ^3^*J*_C-F_ = 12.2 Hz, C-6, Ph-Fu). ESIMS (*m/z*): 323.2 [M + H]^+^. Anal. Calcd. (%) for C_18_H_11_FN_2_OS: C, 67.07; H, 3.44; N, 8.69. Found: C, 66.81; H, 3.74; N, 8.95.

### 2-(5-(4-Chlorophenyl)furan-2-yl)-5-(*p*-tolyl)-1,3,4-thiadiazole (I26)

Yellow solid, m.p. 185–186 °C; IR (KBr) *ν*_*max*_: 2916.81, 1588.09, 1478.17, 1442.49, 1279.54, 1182.15, 1107.90, 1091.51, 1047.16 cm^−1^. ^1^H NMR (400 MHz, CDCl_3_) 2.43 (s, 3H, CH_3_), 6.83 (d, *J* = 3.6 Hz, 1H, FuH), 7.29 (d, *J* = 4.0 Hz, 1H, FuH), 7.31 (d, *J* = 8.4 Hz, 2H, PhH-Thia), 7.41 (d, *J* = 8.4 Hz, 2H, PhH-Fu), 7.70 (d, *J* = 8.8 Hz, 2H, PhH-Fu), 7.90 (d, *J* = 8.0 Hz, 2H, PhH-Thia). ^13^C NMR (100 MHz, CDCl_3_) *δ* 167.36 (C-2, Thia), 157.60 (C-5, Thia), 155.19 (C-5, Fu), 145.19 (C-2, Fu), 141.73 (C-4′, Ph-Thia), 134.34 (C-4, Ph-Fu), 129.91 (2C, C-3′, C-5′, Ph-Thia), 129.13 (2C, C-3, C-5, Ph-Fu), 128.02 (C-1, Ph-Fu), 127.82 (2C, C-2′, C-6′, Ph-Thia), 127.11 (C-1′, Ph-Thia), 125.52 (2C, C-2, C-6, Ph-Fu), 113.70 (C-3, Fu), 108.15 (C-4, Fu), 21.54 (CH_3_). ESIMS (*m/z*): 353.2 [M + H]^+^. Anal. Calcd. (%) for C_19_H_13_ClN_2_OS: C, 64.68; H, 3.71; N, 7.94. Found: C, 64.41; H, 3.98; N, 8.16.

### 2-(5-(2,6-Difluorophenyl)furan-2-yl)-5-(4-methoxyphenyl)-1,3,4-thiadiazole (I27)

Yellow solid, m.p. 178–179 °C; IR (KBr) *ν*_*max*_: 3297.46, 2936.51, 1628.17, 1596.25, 1562.36, 1488.27, 1452.34, 1279.34, 1162.95, 1106.70, 1071.51, 1037.16, 1012.37 cm^−1^. ^1^H NMR (400 MHz, CDCl_3_) 3.89 (s, 3H, OCH_3_), 6.99–7.05 (m, 5H, FuH + 2PhH-Thia + 2PhH-Fu), 7.28–7.32 (m, 1H, PhH-Fu), 7.36 (d, *J* = 3.2 Hz, 1H, FuH), 7.96 (d, *J* = 8.8 Hz, 2H, PhH-Thia). ^13^C NMR (100 MHz, CDCl_3_) *δ* 167.31 (C-2, Thia), 161.96 (C-4′, Ph-Thia), 159.46 (2C, dd, ^1^*J*_C-F_ = 253.2 Hz, ^3^*J*_C-F_ = 6.6 Hz, C-2, C-6, Ph-Fu), 157.37 (C-5, Thia), 146.13 (dd, ^4^*J*_C-F_ = 2.2 Hz, ^4^*J*_C-F_ = 2.3 Hz, C-5, Fu), 145.81 (dd, ^5^*J*_C-F_ = 1.7 Hz, ^5^*J*_C-F_ = 1.7 Hz, C-2, Fu), 129.50 (2C, C-2′, C-6′, Ph-Thia), 129.48 (dd, ^3^*J*_C-F_ = 10.6 Hz, ^3^*J*_C-F_ = 10.5 Hz, C-4, Ph-Fu), 122.65 (C-1′, Ph-Thia), 114.88 (dd, ^3^*J*_C-F_ = 6.4 Hz, ^3^*J*_C-F_ = 6.4 Hz, C-4, Fu), 114.58 (2C, C-3′, C-5′, Ph-Thia), 112.31 (C-3, Fu), 112.18 (2C, dd, ^2^*J*_C-F_ = 19.8 Hz, ^4^*J*_C-F_ = 5.7 Hz, C-3, C-5, Ph-Fu), 108.27 (dd, ^2^*J*_C-F_ = 15.5 Hz, ^2^*J*_C-F_ = 15.6 Hz, C-1, Ph-Fu), 55.50 (OCH_3_). ESIMS (*m/z*): 393.2 [M + Na]^+^. Anal. Calcd. (%) for C_19_H_12_F_2_N_2_O_2_S: C, 61.61; H, 3.27; N, 7.56. Found: C, 61.38; H, 3.54; N, 7.23.

### 2-(4-Chlorophenyl)-5-(5-(2,6-difluorophenyl)furan-2-yl)-1,3,4-thiadiazole (I28)

Yellow solid, m.p. 204–205 °C; IR (KBr) *ν*_*max*_: 3167.46, 2962.43, 1683.77, 1607.38, 1576.65, 1532.27, 1489.57, 1432.14, 1281.34, 1182.55, 1136.51, 1037.16 cm^−1^. ^1^H NMR (400 MHz, CDCl_3_) 7.01–7.06 (m, 3H, FuH + 2PhH-Fu), 7.29–7.33 (m, 1H, PhH-Fu), 7.40 (d, *J* = 3.2 Hz, 1H, FuH), 7.48 (dd, *J* = 2.0, 6.8 Hz, 2H, PhH-Thia), 7.96 (dd, *J* = 2.0, 6.8 Hz, 2H, PhH-Thia). ^13^C NMR (100 MHz, CDCl_3_) *δ* 166.21 (C-2, Thia), 158.28 (C-5, Thia), 159.45 (2C, dd, ^1^*J*_C-F_ = 253.3 Hz, ^3^*J*_C-F_ = 6.5 Hz, C-2, C-6, Ph-Fu), 146.52 (dd, ^4^*J*_C-F_ = 2.3 Hz, ^4^*J*_C-F_ = 2.3 Hz, C-5, Fu), 145.47 (dd, ^5^*J*_C-F_ = 1.8 Hz, ^5^*J*_C-F_ = 1.8 Hz, C-2, Fu), 137.23 (C-4′, Ph-Thia), 129.65 (dd, ^3^*J*_C-F_ = 10.6 Hz, ^3^*J*_C-F_ = 10.6 Hz, C-4, Ph-Fu), 129.49 (2C, C-3′, C-5′, Ph-Thia), 129.05 (2C, C-2′, C-6′, Ph-Thia), 128.43 (C-1′, Ph-Thia), 114.93 (dd, ^3^*J*_C-F_ = 6.5 Hz, ^3^*J*_C-F_ = 6.4 Hz, C-4, Fu), 112.31 (C-3, Fu), 112.20 (2C, dd, ^2^*J*_C-F_ = 19.9 Hz, ^4^*J*_C-F_ = 5.7 Hz, C-3, C-5, Ph-Fu), 108.14 (dd, ^2^*J*_C-F_ = 15.5 Hz, ^2^*J*_C-F_ = 15.4 Hz, C-1, Ph-Fu). ESIMS (*m/z*): 375.1 [M + H]^+^. Anal. Calcd. (%) for C_18_H_9_ClF_2_N_2_OS: C, 57.68; H, 2.42; N, 7.47. Found: C, 57.92; H, 2.21; N, 7.69.

### 2-(2-Chlorophenyl)-5-(5-(2-nitrophenyl)furan-2-yl)-1,3,4-thiadiazole (I29)

Brown solid, m.p. 148–149 °C; IR (KBr) *ν*_*max*_: 2928.35, 1617.63, 1528.54, 1482.13, 1451.37, 1427.33, 1338.32, 1319.32, 1268.91, 1185.43, 1109.62, 1022.24, 1018.51 cm^−1^. ^1^H NMR (400 MHz, CDCl_3_) 6.87 (d, *J* = 4.0 Hz, 1H, FuH), 7.36 (d, *J* = 4.0 Hz, 1H, FuH), 7.44–7.46 (m, 2H, PhH-Thia), 7.50–7.57 (m, 2H, PhH-Thia + PhH-Fu), 7.65–7.69 (m, 1H, PhH-Fu), 7.79 (dd, *J* = 0.8, 8.0 Hz, 1H, PhH-Fu), 7.84 (dd, *J* = 1.0, 8.0 Hz, 1H, PhH-Fu), 8.37–8.39 (m, 1H, PhH-Thia). ^13^C NMR (100 MHz, CDCl_3_) *δ* 162.93 (C-2, Thia), 159.14 (C-5, Thia), 150.67 (C-5, Fu), 147.64 (C-2, Ph-Fu), 146.40 (C-2, Fu), 132.55 (C-1′, Ph-Thia), 132.22 (C-4, Ph-Fu), 131.72 (C-4′, Ph-Thia), 131.18 (C-3′, Ph-Thia), 130.61 (C-6′, Ph-Thia), 129.41 (C-5, Ph-Fu), 129.30 (C-3, Ph-Fu), 128.66 (C-2′, Ph-Thia), 127.40 (C-5′, Ph-Thia), 124.19 (C-6, Ph-Fu), 123.04 (C-1, Ph-Fu), 113.44 (C-3, Fu), 112.35 (C-4, Fu). ESIMS (*m/z*): 384.1 [M + H]^+^. Anal. Calcd. (%) for C_18_H_10_ClN_3_O_3_S: C, 56.33; H, 2.63; N, 10.95. Found: C, 56.08; H, 2.39; N, 11.14.

### 2-(5-(2-Nitrophenyl)furan-2-yl)-5-(*p*-tolyl)-1,3,4-thiadiazole (I30)

Yellow solid, m.p. 146–147 °C; IR (KBr) *ν*_*max*_: 1642.52, 1521.51, 1474.31, 1446.39, 1417.45, 1339.23, 1301.39, 1278.32, 1164.34, 1117.36, 1021.59 cm^−1^. ^1^H NMR (400 MHz, CDCl_3_) 2.43 (s, 3H, CH_3_), 6.87 (d, *J* = 4.0 Hz, 1H, FuH), 7.30 (d, *J* = 8.4 Hz, 2H, PhH-Thia), 7.32 (d, *J* = 4.0 Hz, 1H, FuH), 7.49–7.53 (m, 1H, PhH-Fu), 7.64–7.67 (m, 1H, PhH-Fu), 7.77–7.82 (m, 2H, PhH-Fu), 7.90 (d, *J* = 8.0 Hz, 2H, PhH-Thia). ^13^C NMR (100 MHz, CDCl_3_) *δ* 167.91 (C-2, Thia), 157.24 (C-5, Thia), 150.51 (C-5, Fu), 147.62 (C-2, Ph-Fu), 146.66 (C-2, Fu), 141.80 (C-4′, Ph-Thia), 132.14 (C-4, Ph-Fu), 129.90 (2C, C-3′, C-5′, Ph-Thia), 129.34 (C-5, Ph-Fu), 129.14 (C-3, Ph-Fu), 127.91 (2C, C-2′, C-6′, Ph-Thia), 127.06 (C-1′, Ph-Thia), 124.18 (C-6, Ph-Fu), 122.99 (C-1, Ph-Fu), 112.95 (C-3, Fu), 112.24 (C-4, Fu), 21.55 (CH_3_). ESIMS (*m/z*): 364.1 [M + H]^+^. Anal. Calcd. (%) for C_19_H_13_N_3_O_3_S: C, 62.80; H, 3.61; N, 11.56. Found: C, 63.02; H, 3.83; N, 11.39.

### 2-(4-Methoxyphenyl)-5-(5-(3-nitrophenyl)furan-2-yl)-1,3,4-thiadiazole (I31)

Yellow solid, m.p. 200–201 °C; IR (KBr) *ν*_*max*_: 3096.43, 1617.56, 1516.83, 1497.91, 1458.23, 1341.53, 1310.35, 1276.39, 1163.47, 11091.6, 1031.42 cm^−1^. ^1^H NMR (400 MHz, CDCl_3_) 3.90 (s, 3H, OCH_3_), 7.00–7.03 (m, 3H, FuH + 2PhH-Thia), 7.33 (d, *J* = 3.6 Hz, 1H, FuH), 7.61–7.65 (m, 1H, PhH-Fu), 7.97 (d, *J* = 8.8 Hz, 2H, PhH-Thia), 8.06–8.09 (m, 1H, PhH-Fu), 8.17–8.20 (m, 1H, PhH-Fu), 8.50–8.58 (m, 1H, PhH-Fu). ^13^C NMR (100 MHz, CDCl_3_) *δ* 167.44 (C-2, Thia), 162.09 (C-4′, Ph-Thia), 156.80 (C-5, Thia), 153.40 (C-5, Fu), 148.78 (C-3, Ph-Fu), 146.27 (C-2, Fu), 131.12 (C-1, Ph-Fu), 130.02 (C-6, Ph-Fu), 129.68 (C-5, Ph-Fu), 129.54 (2C, C-2′, C-6′, Ph-Thia), 122.76 (C-4, Ph-Fu), 122.42 (C-1′, Ph-Thia), 118.94 (C-2, Ph-Fu), 114.64 (2C, C-3′, C-5′, Ph-Thia), 113.40 (C-3, Fu), 109.85 (C-4, Fu), 55.52 (OCH_3_). ESIMS (*m/z*): 380.2 [M + H]^+^. Anal. Calcd. (%) for C_19_H_13_N_3_O_4_S: C, 60.15; H, 3.45; N, 11.08. Found: C, 59.89; H, 3.68; N, 11.21.

### 2-(5-(2,6-Difluorophenyl)furan-2-yl)-5-phenyl-1,3,4-thiadiazole (I32)

Yellow solid, m.p. 196–197 °C; IR (KBr) *ν*_*max*_: 3147.76, 2982.53, 1673.37, 1601.28, 1566.45, 1522.16, 1484.25, 1421.78, 1280.54, 1182.43, 1135.69 cm^−1^. ^1^H NMR (400 MHz, CDCl_3_) 7.00–7.05 (m, 3H, FuH + 2PhH-Fu), 7.28–7.32 (m, 1H, PhH-Fu), 7.39 (d, *J* = 3.6 Hz, 1H, FuH), 7.49–7.52 (m, 3H, PhH-Thia), 8.00–8.03 (m, 2H, PhH-Thia). ^13^C NMR (100 MHz, CDCl_3_) *δ* 167.50 (C-2, Thia), 159.46 (2C, dd, ^1^*J*_C-F_ = 253.2 Hz, ^3^*J*_C-F_ = 6.5 Hz, C-2, C-6, Ph-Fu), 158.10 (C-5, Thia), 146.35 (dd, ^4^*J*_C-F_ = 2.3 Hz, ^4^*J*_C-F_ = 2.3 Hz, C-5, Fu), 145.68 (dd, ^5^*J*_C-F_ = 1.7 Hz, ^5^*J*_C-F_ = 1.7 Hz, C-2, Fu), 131.15 (C-1′, Ph-Thia), 129.95 (C-4′, Ph-Thia), 129.57 (dd, ^3^*J*_C-F_ = 10.6 Hz, ^3^*J*_C-F_ = 10.5 Hz, C-4, Ph-Fu), 129.21 (2C, C-2′, C-6′, Ph-Thia), 127.93 (2C, C-3′, C-5′, Ph-Thia), 114.91 (dd, ^3^*J*_C-F_ = 6.4 Hz, ^3^*J*_C-F_ = 6.4 Hz, C-4, Fu), 112.30 (C-3, Fu), 112.20 (2C, dd, ^2^*J*_C-F_ = 19.9 Hz, ^4^*J*_C-F_ = 5.7 Hz, C-3, C-5, Ph-Fu), 108.21 (dd, ^2^*J*_C-F_ = 15.5 Hz, ^2^*J*_C-F_ = 15.5 Hz, C-1, Ph-Fu). ESIMS (*m/z*): 341.2 [M + H]^+^. Anal. Calcd. (%) for C_18_H_10_F_2_N_2_OS: C, 63.52; H, 2.96; N, 8.23. Found: C, 63.37; H, 2.75; N, 8.46.

### Bioassays

#### *In vitro* fungicidal activity

*In vitro* fungicidal activity of the title compounds against *Phytophthora infestans*, *Valsa mali*, *Phomopsis aspamgi*, *Cladosporium fulvum* Cke., *Alternaria tenuis* Nees and other twenty panthogenic fungi listed in Table 4 were evaluated using mycelium growth rate test^22^,^30^. The tested compounds were dissolved in DMSO (dimethyl sulfoxide) and mixed with sterile molten potato dextrose agar to a final concentration of 50 μg mL^−1^. Commercial fungicides pyrimorph and hymexazol were used as controls against the above mentioned fungal pathogens under the same conditions. Three replicates were performed. The relative inhibitory rate of title compounds compared to blank control was calculated *via* the following equation (1):


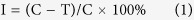


In which, I stands for the rate of inhibition (%), C is the diameter of mycelia in the blank control test (in mm), and T is the diameter of mycelia in the presence of tested compounds (in mm). The EC50 values of title compounds were evaluated using logit analysis (Table 2). EC50 results were analyzed using the statistical data processing system (DPS, 10.15, Zhejiang, China).

#### *In vivo* antifungal activity

Using the pot culture test^22^,^28^, *in vivo* antifungal activities of the title compounds against *P. infestans*, *V. mali*, *P. aspamgi* and *C. fulvum* Cke. were evaluated in greenhouse along with commercial fungicides pyrimorph and hymexazol as controls.

The culture plates were cultivated at 24 ± 1 °C. Soaking cucumber seeds in water for 2 h at 50 ^o^C, and then, keeping the seeds moist for 24 h at 28 ^o^C in an incubator. When the radicles were 0.5 cm, the seeds were grown in plastic pots containing a 1:1 (v/v) mixture of vermiculite and peat. Cucumber plants used for inoculations were at the stage of two seed leaves.

Tested compounds were confected to 2.5% EC (emulsifiable cocentration) formulations, in which pesticide emulsifier 600 (2.125%) and pesticide emulsifier 500 (0.375%) were the additives, DMSO (0.1%) was the solvent, and xylene was the co-solvent. The formulation was diluted to 500 μg mL^−1^ with water. The pathogenic fungi were inoculated on the surface of seed leaves and then the solution of title compounds was sprayed with a hand sprayer, respectively. Three replicates for each treatment were applied. After inoculation, the plants were maintained at 24 ± 1 ^o^C and above 80% relative humidity.

When the untreated cucumber plant (blank control) fully developed symptoms, the fungicidal activity was assessed. The area of inoculated leaves covered by disease symptoms was evaluated and compared to that of untreated ones to determine the average disease index. The relative control efficacy of compounds compared to the blank assay was calculated *via* the following equation (2):


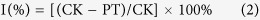


where I is relative control efficacy, CK is the average disease index during the blank assay and PT is the average disease index after treatment during testing. The results are shown in Table 3.

### Electron microscopy^42^,^43^

#### Scanning electron microscopy (SEM)

Mycelial tip (5 mm) of *P. capsici* from an actively growing colony on PDA medium, which were treated by **I18** at 50 μg mL^−1^, were cut from the edge of the colony after cultured for 72 h. The tips were treated with 4% glutaraldehyde at 4 ^o^C, and then, rinsed with 0.1 M phosphate buffer (pH 7.3), and fixed with 1% w/v osmium tetraoxide solution. The mycelial tips were dehydrated using a series of acetone solutions in the order of concentration 30, 50, 70, 80, and 90% anhydrous acetone, after rinsed with 0.1 M phosphate buffer three times. After completing the processes of drying at critical point, mounting, and gold spraying, the mycelial tip was examined by a scanning electron microscope (S-3400N, Hitachi, Nissei Sanyo, Japan) with an accelerating voltage of 18–20 kV.

#### Transmission electron microscopy (TEM)

The mycelial tip was prepared according to the same method mentioned above. After dehydrating and embedding in Epon 112, thin sections were cut and double-stained with uranyl acetate and lead citrate. The grids were examined with a JEOL-1230 (JEOL, Tokyo, Japan) transmission electron microscope.

## Additional Information

**How to cite this article**: Cui, Z.-N. *et al.* Synthesis and fungicidal activity of novel 2,5-disubstituted-1,3,4- thiadiazole derivatives containing 5-phenyl-2-furan. *Sci. Rep.* **6**, 20204; doi: 10.1038/srep20204 (2016).

## Supplementary Material

Supplementary Information

## Figures and Tables

**Table 1:** Comparison Between Conventional Heating Method and Microwave Assisted Method for Synthesis of Title Compounds in Terms of Time and Yield.

Compd.	Conventional		Microwave	
	Time (min)	Yield (%)	Time (min)	Yield (%)
**I1**	360	68.9	15	81.6
**I2**	360	66.4	15	78.1
**I3**	360	71.2	15	85.2
**I4**	360	62.7	15	81.0
**I5**	360	65.2	15	78.5
**I6**	360	67.1	15	79.4
**I7**	360	73.4	15	82.4
**I8**	360	70.8	15	85.7
**I9**	360	70.1	15	78.9
**I10**	360	68.0	15	79.8
**I11**	360	75.4	15	86.1
**I12**	360	73.6	15	87.5
**I13**	360	69.7	15	85.4
**I14**	360	70.1	15	79.9
**I15**	360	69.4	15	78.2
**I16**	360	65.1	15	74.5
**I17**	360	62.8	15	79.6
**I18**	360	66.4	15	78.1
**I19**	360	63.9	15	74.3
**I20**	360	62.1	15	73.8
**I21**	360	70.5	15	85.6
**I22**	420	71.6	15	84.1
**I23**	420	65.4	15	74.5
**I24**	420	69.8	15	78.1
**I25**	420	66.9	15	76.7
**I26**	420	69.1	15	84.9
**I27**	300	75.9	15	85.0
**I28**	420	76.4	15	86.4
**I29**	360	65.0	15	82.7
**I30**	360	72.1	15	80.6
**I31**	300	71.0	15	82.0
**I32**	300	68.2	15	79.4

**Table 2:** *In Vitro* Fungicidal Activities of Title Compounds against Five Fungus Species.

Compd.	R_1_	R_2_	EC_50_ (μg mL^−1^)				
			*P. infestans*	*V. mali*	*P. aspamgi*	*C. fulvum*	*A. tenuis*
**I1**	4-OCH_3_	4-NO_2_	22.1	61.2	66.5	68.4	97.2
**I2**	4-Br	2-Cl	61.2	50.1	45.2	19.7	154.6
**I3**	4-Cl	4-OCH_3_	51.8	71.5	51.3	42.1	112.5
**I4**	2-OCH_3_	4-Cl	78.2	75.4	152.1	157.9	98.9
**I5**	3-Cl	4-Cl	81.4	76.3	82.1	21.5	171.5
**I6**	4-OCH_3_	4-Br	91.7	48.1	78.5	69.1	82.9
**I7**	4-Cl	2-Cl	101.8	121.4	71.5	78.5	125.8
**I8**	2-Cl	2-Cl	78.6	62.1	123.4	79.4	52.7
**I9**	4-OEt	2-Cl	69.2	63.5	120.5	68.3	124.9
**I10**	H	2-Cl	7.4	65.7	111.8	178.4	76.1
**I11**	4-Cl	2,4-di-F	31.7	171.8	62.4	62.7	62.1
**I12**	2-Cl	2,4-di-F	84.5	68.4	22.8	65.1	86.7
**I13**	4-CH_3_	2,4-di-F	74.8	74.8	42.1	82.6	89.1
**I14**	4-OCH_3_	2,4-di-F	69.4	79.5	45.8	64.2	96.4
**I15**	3-CH_3_	4-F	32.1	152.4	142.1	44.9	156.8
**I16**	2-Cl	3-F	45.2	45.1	78.6	157.3	175.9
**I17**	4-CH_3_	4-F	112.5	123.4	41.2	163.7	71.2
**I18**	H	4-F	5.7	9.7	21.7	21.4	5.8
**I19**	4-Cl	H	4.1	45.1	51.8	81.5	182.1
**I20**	2-Cl	H	67.1	41.3	23.1	22.8	96.2
**I21**	3-CH_3_	4-OCH_3_	56.5	20.4	50.2	51.2	152.6
**I22**	4-Cl	4-CH_3_	111.5	50.8	50.8	62.7	92.5
**I23**	4-OCH_3_	2-F	74.2	75.3	38.9	86.1	154.9
**I24**	3-CH_3_	2-F	59.5	74.9	98.7	125.4	136.4
**I25**	H	2-F	8.4	97.2	74.8	32.8	71.6
**I26**	4-CH_3_	4-Cl	51.2	84.6	99.7	81.4	70.5
**I27**	4-OCH_3_	2,6-di-F	56.4	162.4	142.7	127.9	82.4
**I28**	4-Cl	2,6-di-F	31.4	62.4	77.5	62.8	86.3
**I29**	2-Cl	2-NO_2_	55.7	51.1	24.7	51.2	62.1
**I30**	4-CH_3_	2-NO_2_	66.7	98.4	35.6	38.9	128.7
**I31**	4-OCH_3_	3-NO_2_	18.1	78.5	38.4	88.1	159.8
**I32**	H	2,6-di-F	55.4	65.4	78.9	69.4	102.7
pyrimorph			25.2	32.5	27.8	35.4	17.3
hymexazol			29.1	10.9	11.7	15.3	7.4

**Table 3:** *In Vivo* Fungicidal Activities of Title Compounds against Four Fungus Species at 500 μg mL^−1^.

Compd.	R_1_	R_2_	control efficacy (%)			
			*P. infestans*	*V. mali*	*P. aspamgi*	*C. fulvum*
**I1**	4-OCH_3_	4-NO_2_	68.65 ± 1.28	58.11 ± 0.59	54.14 ± 1.56	39.11 ± 1.25
**I2**	4-Br	2-Cl	39.15 ± 1.56	56.87 ± 1.62	64.35 ± 1.56	59.35 ± 1.22
**I3**	4-Cl	4-OCH_3_	44.34 ± 1.22	36.09 ± 1.02	39.58 ± 1.00	35.56 ± 2.05
**I4**	2-OCH_3_	4-Cl	12.61 ± 0.42	22.15 ± 1.24	15.59 ± 0.97	6.56 ± 0.61
**I5**	3-Cl	4-Cl	10.22 ± 1.21	18.12 ± 1.05	33.12 ± 1.02	60.11 ± 1.25
**I6**	4-OCH_3_	4-Br	7.89 ± 0.45	56.25 ± 2.13	39.11 ± 1.53	21.45 ± 0.58
**I7**	4-Cl	2-Cl	8.35 ± 0.65	10.24 ± 1.38	39.13 ± 1.61	41.46 ± 1.05
**I8**	2-Cl	2-Cl	7.32 ± 0.71	30.56 ± 1.12	9.57 ± 0.39	39.22 ± 1.12
**I9**	4-OEt	2-Cl	7.90 ± 0.75	20.50 ± 0.89	2.61 ± 0.12	61.16 ± 0.89
**I10**	H	2-Cl	83.85 ± 1.85	28.84 ± 1.98	4.38 ± 0.22	14.13 ± 0.51
**I11**	4-Cl	2,4-di-F	67.28 ± 1.13	5.67 ± 1.05	40.02 ± 1.02	54.25 ± 1.60
**I12**	2-Cl	2,4-di-F	15.00 ± 0.23	44.11 ± 2.02	62.58 ± 2.06	41.48 ± 1.05
**I13**	4-CH_3_	2,4-di-F	8.99 ± 0.52	24.25 ± 1.05	63.25 ± 1.62	45.24 ± 1.51
**I14**	4-OCH_3_	2,4-di-F	9.35 ± 0.65	30.19 ± 1.15	57.13 ± 2.02	43.97 ± 1.25
**I15**	3-CH_3_	4-F	56.48 ± 2.03	15.09 ± 2.02	7.78 ± 1.03	53.47 ± 1.83
**I16**	2-Cl	3-F	37.24 ± 1.14	47.94 ± 1.11	36.22 ± 1.21	9.54 ± 0.69
**I17**	4-CH_3_	4-F	8.25 ± 0.75	10.78 ± 1.25	51.20 ± 1.08	9.58 ± 0.56
**I18**	H	4-F	84.21 ± 1.58	68.54 ± 2.10	61.86 ± 1.72	77.14 ± 2.02
**I19**	4-Cl	H	87.15 ± 2.02	43.85 ± 0.95	47.09 ± 1.58	25.29 ± 1.05
**I20**	2-Cl	H	12.20 ± 1.15	53.57 ± 1.57	62.56 ± 1.85	71.55 ± 1.20
**I21**	3-CH_3_	4-OCH_3_	15.51 ± 1.05	65.11 ± 1.12	50.91 ± 1.35	49.54 ± 1.20
**I22**	4-Cl	4-CH_3_	16.11 ± 1.13	42.98 ± 1.04	53.11 ± 1.11	34.25 ± 1.04
**I23**	4-OCH_3_	2-F	11.25 ± 1.21	28.15 ± 1.10	48.25 ± 2.25	26.11 ± 0.65
**I24**	3-CH_3_	2-F	10.00 ± 1.20	38.51 ± 1.26	16.28 ± 0.86	11.03 ± 1.01
**I25**	H	2-F	80.18 ± 2.01	21.19 ± 1.01	33.21 ± 0.99	61.97 ± 2.02
**I26**	4-CH_3_	4-Cl	11.33 ± 1.65	36.75 ± 1.22	14.25 ± 0.52	22.25 ± 1.25
**I27**	4-OCH_3_	2,6-di-F	13.18 ± 0.25	5.95 ± 0.81	11.57 ± 0.61	12.22 ± 0.58
**I28**	4-Cl	2,6-di-F	34.12 ± 1.02	38.14 ± 0.68	31.85 ± 1.26	39.94 ± 1.05
**I29**	2-Cl	2-NO_2_	19.24 ± 1.05	42.18 ± 1.42	64.11 ± 1.27	49.11 ± 1.24
**I30**	4-CH_3_	2-NO_2_	14.22 ± 1.04	16.52 ± 1.05	49.28 ± 1.00	45.78 ± 1.05
**I31**	4-OCH_3_	3-NO_2_	71.66 ± 1.42	18.22 ± 1.24	55.57 ± 2.14	36.11 ± 1.20
**I32**	H	2,6-di-F	31.95 ± 1.23	18.82 ± 1.15	23.77 ± 1.02	38.98 ± 2.01
pyrimorph			77.15 ± 1.84	64.22 ± 1.24	60.21 ± 1.53	46.21 ± 1.19
hymexazol			64.27 ± 1.72	78.98 ± 2.01	83.15 ± 2.17	88.28 ± 2.14

**Table 4:** *In Vitro* Fungicidal Activities of Compound I18 Against Twenty Fungus Species at 50 μg mL^−1^.

Fungus	I18	pyrimorph	hymexazol
*H. s.*	88.34 ± 1.75	48.61 ± 1.12	72.50 ± 1.26
*F. m*.	78.57 ± 1.62	74.71 ± 2.05	50.48 ± 1.10
*F. g.*	88.61 ± 2.02	60.59 ± 1.15	38.96 ± 0.87
*P. o.*	66.41 ± 1.04	55.49 ± 1.14	79.85 ± 1.37
*E. t.*	58.25 ± 1.15	37.94 ± 1.11	58.25 ± 1.02
*F. c.*	78.99 ± 1.16	50.78 ± 1.25	95.66 ± 2.03
*A. a.*	96.89 ± 2.38	68.79 ± 0.99	95.76 ± 1.61
*T. r.*	27.30 ± 0.64	53.05 ± 0.95	98.18 ± 2.19
*P. o.*	79.75 ± 1.59	63.87 ± 1.07	75.00 ± 0.92
*M. a*.	98.90 ± 2.01	60.57 ± 1.14	57.60 ± 0.72
*G. m.*	91.90 ± 1.75	67.89 ± 0.78	89.56 ± 1.02
*C. g.*	96.16 ± 2.07	40.58 ± 0.82	49.83 ± 0.86
*F. o.*	92.71 ± 2.05	42.66 ± 0.74	88.00 ± 1.62
*B. c*.	30.78 ± 0.72	68.78 ± 2.19	74.44 ± 1.43
*S. f.*	90.37 ± 1.42	79.97 ± 1.24	78.97 ± 1.03
*P. m.*	48.71 ± 1.11	43.70 ± 0.91	93.02 ± 1.11
*C. o.*	93.19 ± 1.85	68.89 ± 1.77	59.34 ± 0.68
*A. d.*	97.70 ± 2.10	79.27 ± 1.81	97.70 ± 1.30
*R. s.*	88.89 ± 1.95	24.54 ± 0.76	79.89 ± 1.60
*P. c.*	92.72 ± 1.43	43.21 ± 0.68	83.75 ± 2.00

H. s.: Helminthosporium sativum; F. m.: Fusarium monihforme Sheld.; F. g.: Fusarium graminearum; P. o.: Pyricularia oryzae; E. t.: Exserohilum turcicum = Helminthosporium turcicum; F. c.: Fusarium coeruleum; A. a.: Alternaria alternate; T. r.: Trichothecium roseum (Bvll.) Link; P. o.: Phomopsis obscurans; M. a.: Monilinia ariae (Schellenb.) Whetz.; G. m.: Gloeosporium musarum; C. g.: Colletotrichum gloeosporioides Penz.; F. o.: Fusarium oxysporum; B. c.: Botrytis cinerea Pers.; S. f.: Sphaerotheca fuliginea; P. m.: Phytophthora melonis; C. o.: Colletotrichum orbiculare; A. d.: Alternaria dauci (Kühn.) Groves et Skolko; R. s.: Rhizoctonia solanii; P. c.: Phytophthora capsici.
