# Supplementary material for: Synthesis and fungicidal activity of novel 2,5-disubstituted-1,3,4- thiadiazole derivatives containing 5-phenyl-2-furan
Source: Sci Rep. 2016 Jan 29;6:20204. doi: 10.1038/srep20204 (PMC4731749; doi:10.1038/srep20204)
Supplement: Supplementary Information [file srep20204-s1.doc]

**Synthesis and fungicidal activity of novel 2,5-disubstituted-1,3,4- thiadiazole derivatives containing 5-phenyl-2-furan**

Zi-Ning Cui^1,*^, Ya-Sheng Li^1^, De-Kun Hu^1^, Hao Tian^1^, Jia-Zhen Jiang^2^, Yuan Wang^1^, Xiao-Jing Yan^3,*^

^1^ Guangdong Province Key Laboratory of Microbial Signals and Disease Control, Department of Plant Pathology, South China Agricultural University, Guangzhou 510642, China.

^2^ Department of Applied Chemistry, College of Science, China Agricultural University, Beijing 100193, China

^3^ Institute of Plant Protection, Chinese Academy of Agricultural Sciences, Beijing 100193, China.

*Corresponding author Tel: +86 20 85288229; Fax: +86 20 85288229; E-mail: [ziningcui@scau.edu.cn](mailto:ziningcui@scau.edu.cn) (Z. C.); [yanxiaojing@caas.cn](mailto:yanxiaojing@caas.cn) (X. Y.)

**Figure S1-1.** ^1^H NMR spectrum of compound **I1**

**Figure S1-2.** ^13^C NMR spectrum of compound **I1**

**Figure S2-1.** ^1^H NMR spectrum of compound **I2**

**Figure S2-2.** ^13^C NMR spectrum of compound **I2**

**Figure S3-1.** ^1^H NMR spectrum of compound **I3**

**Figure S3-2.** ^13^C NMR spectrum of compound **I3**

**Figure S4-1.** ^1^H NMR spectrum of compound **I4**

**Figure S4-2.** ^13^C NMR spectrum of compound **I4**

**Figure S5-1.** ^1^H NMR spectrum of compound **I5**

**Figure S5-2.** ^13^C NMR spectrum of compound **I5**

**Figure S6-1.** ^1^H NMR spectrum of compound **I6**

**Figure S6-2**. ^13^C NMR spectrum of compound **I6**

**Figure S7-1.** ^1^H NMR spectrum of compound **I7**

**Figure S7-2.** ^13^C NMR spectrum of compound **I7**

**Figure S8-1.** ^1^H NMR spectrum of compound **I8**

**Figure S8-2.** ^13^C NMR spectrum of compound **I8**

**Figure S9-1.** ^1^H NMR spectrum of compound **I9**

**Figure S9-2.** ^13^C NMR spectrum of compound **I9**

**Figure S10-1.** ^1^H NMR spectrum of compound **I10**

**Figure S10-2.** ^13^C NMR spectrum of compound **I10**

**Figure S11-1.** ^1^H NMR spectrum of compound **I11**

**Figure S11-2.** ^13^C NMR spectrum of compound **I11**

**Figure S12-1.** ^1^H NMR spectrum of compound **I12**

**Figure S12-2.** ^13^C NMR spectrum of compound **I12**

**Figure S13-1.** ^1^H NMR spectrum of compound **I13**

**Figure S13-2.** ^13^C NMR spectrum of compound **I13**

**Figure S14-1.** ^1^H NMR spectrum of compound **I14**

**Figure S14-2.** ^13^C NMR spectrum of compound **I14**

**Figure S15-1.** ^1^H NMR spectrum of compound **I15**

**Figure S15-2.** ^13^C NMR spectrum of compound **I15**

**Figure S16-1.** ^1^H NMR spectrum of compound **I16**

**Figure S16-2.** ^13^C NMR spectrum of compound **I16**

**Figure S17-1.** ^1^H NMR spectrum of compound **I17**

**Figure S17-2.** ^13^C NMR spectrum of compound **I17**

**Figure S18-1.** ^1^H NMR spectrum of compound **I18**

**Figure S18-2.** ^13^C NMR spectrum of compound **I18**

**Figure S19-1.** ^1^H NMR spectrum of compound **I19**

**Figure S19-2**. ^13^C NMR spectrum of compound **I19**

**Figure S20-1.** ^1^H NMR spectrum of compound **I20**

**Figure S20-2.** ^13^C NMR spectrum of compound **I20**

**Figure S21-1.** ^1^H NMR spectrum of compound **I21**

**Figure S21-2.** ^13^C NMR spectrum of compound **I21**

**Figure S22-1.** ^1^H NMR spectrum of compound **I22**

**Figure S22-2.** ^13^C NMR spectrum of compound **I22**

**Figure S23-1.** ^1^H NMR spectrum of compound **I23**

**Figure S23-2.** ^13^C NMR spectrum of compound **I23**

**Figure S24-1.** ^1^H NMR spectrum of compound **I24**

**Figure S24-2.** ^13^C NMR spectrum of compound **I24**

**Figure S25-1.** ^1^H NMR spectrum of compound **I25**

**Figure S25-2.** ^13^C NMR spectrum of compound **I25**

**Figure S26-1.** ^1^H NMR spectrum of compound **I26**

**Figure S26-2.** ^13^C NMR spectrum of compound **I26**

**Figure S27-1.** ^1^H NMR spectrum of compound **I27**

**Figure S27-2.** ^13^C NMR spectrum of compound **I27**

**Figure S28-1.** ^1^H NMR spectrum of compound **I28**

**Figure S28-2.** ^13^C NMR spectrum of compound **I28**

**Figure S29-1.** ^1^H NMR spectrum of compound **I29**

**Figure S29-2.** ^13^C NMR spectrum of compound **I29**

**Figure S30-1.** ^1^H NMR spectrum of compound **I30**

**Figure S30-2.** ^13^C NMR spectrum of compound **I30**

**Figure S31-1.** ^1^H NMR spectrum of compound **I31**

**Figure S31-2.** ^13^C NMR spectrum of compound **I31**

**Figure S32-1.** ^1^H NMR spectrum of compound **I32**

**Figure S32-2.** ^13^C NMR spectrum of compound **I32**
